# cGMP via PKG activates 26S proteasomes and enhances degradation of proteins, including ones that cause neurodegenerative diseases

**DOI:** 10.1073/pnas.2003277117

**Published:** 2020-06-08

**Authors:** Jordan J. S. VerPlank, Sylwia D. Tyrkalska, Angeleen Fleming, David C. Rubinsztein, Alfred L. Goldberg

**Affiliations:** ^a^Department of Cell Biology, Blavatnik Institute, Harvard Medical School, Boston, MA 02115;; ^b^Department of Medical Genetics, Cambridge Institute for Medical Research, University of Cambridge, CB2 0XY Cambridge, United Kingdom;; ^c^United Kingdom Dementia Research Institute, University of Cambridge, CB2 0XY Cambridge, United Kingdom;; ^d^Department of Physiology, Development, and Neuroscience, University of Cambridge, CB2 3DY Cambridge, United Kingdom

**Keywords:** cGMP, protein kinase G, proteasome phosphorylation, protein degradation

## Abstract

Because raising cAMP enhances 26S proteasome activity and the degradation of cell proteins, including the selective breakdown of misfolded proteins, we investigated whether agents that raise cGMP may also regulate protein degradation. Treating various cell lines with inhibitors of phosphodiesterase 5 or stimulators of soluble guanylyl cyclase rapidly enhanced multiple proteasome activities and cellular levels of ubiquitinated proteins by activating protein kinase G (PKG). PKG stimulated purified 26S proteasomes by phosphorylating a different 26S component than is modified by protein kinase A. In cells and cell extracts, raising cGMP also enhanced within minutes ubiquitin conjugation to cell proteins. Raising cGMP, like raising cAMP, stimulated the degradation of short-lived cell proteins, but unlike cAMP, also markedly increased proteasomal degradation of long-lived proteins (the bulk of cell proteins) without affecting lysosomal proteolysis. We also tested if raising cGMP, like cAMP, can promote the degradation of mutant proteins that cause neurodegenerative diseases. Treating zebrafish models of tauopathies or Huntington’s disease with a PDE5 inhibitor reduced the levels of the mutant huntingtin and tau proteins, cell death, and the resulting morphological abnormalities. Thus, PKG rapidly activates cytosolic proteasomes, protein ubiquitination, and overall protein degradation, and agents that raise cGMP may help combat the progression of neurodegenerative diseases.

## Significance

Most studies of the regulation of proteolysis by the ubiquitin proteasome system have focused on the control of ubiquitination. However, it is now clear that the activity of the 26S proteasome and rates of protein degradation in cells are also tightly regulated through proteasome phosphorylation. Here we demonstrate that agents that raise cGMP and activate cGMP-dependent protein kinase (e.g., widely used phosphodiesterase 5 inhibitors) stimulate proteasome activities and intracellular proteolysis without affecting autophagy. Furthermore, we showed that raising cGMP reduced the levels of the disease-causing mutant tau in a zebrafish model by increasing its degradation, and also decreased the associated morphological abnormalities. Thus, activating the proteasome via cGMP is a promising strategy to prevent the progression of neurodegenerative diseases.

In mammalian cells, the great majority of proteins are degraded by the ubiquitin proteasome system (UPS) (1). These proteins are targeted for degradation by linkage to a ubiquitin chain and are then hydrolyzed by the 26S proteasome. Because of the very large number of enzymes specifically involved in ubiquitinating different substrates, and the many cellular processes that are regulated by ubiquitination of key proteins, it is generally assumed that a protein’s rate of degradation is determined solely by its rate of ubiquitination. However, there is growing evidence that protein half-lives are also influenced by changes in proteasome activity, as occur in various physiological and pathological conditions (1, 2). One form of proteasome regulation that has been recently established to be physiologically important and therapeutically promising is the phosphorylation of proteasome subunits (2). A number of kinases have been reported to alter proteasome activities (2), including CAMKII (3), ASK1 (4, 5), Aurora B (6), p38 MAPK (5, 7), PIM (8), and protein kinase G (PKG) (9, 10), although only two have been shown to have clear effects on intracellular protein degradation and to enhance proteasome function via subunit phosphorylation: DYRK2 (11, 12) and protein kinase A (PKA) (13–15).

cAMP and PKA were shown to increase the ability of purified 26S proteasomes to hydrolyze polyubiquitinated proteins, ATP, and small peptides via phosphorylation of Serine 14 in Rpn6, a subunit of the 19S regulatory complex (13, 15). Activating PKA with pharmacological agents that increase cAMP synthesis by adenylyl cyclases or inhibit its breakdown by phosphodiesterase 4 stimulated the selective degradation of short-lived cell proteins, which include misfolded and many regulatory proteins, but not the long-lived cell constituents, which comprise the bulk of cell proteins (13). A similar activation of proteasomes and protein degradation in vivo by PKA also occurs in response to various endocrine stimuli that act by raising cAMP levels (15). For example, proteasome activity, Rpn6-S14 phosphorylation, and the breakdown of short-lived proteins are all enhanced in mouse hepatocytes after exposure to glucagon or epinephrine and in kidney cells by vasopressin (15). Proteasome activity also increases by this mechanism in livers and skeletal muscles of mice when cAMP levels increased during a brief fast and in skeletal muscles of humans and rats upon intense exercise (15). Furthermore, the ability of PKA to stimulate the degradation of misfolded proteins has therapeutic promise because pharmacological agents that raise cAMP enhanced the capacity of cultured cells (13) and mouse brains (14, 16) to destroy misfolded proteins, such as mutant forms of tau that cause frontotemporal dementia. In the present study, we demonstrate that raising cGMP and activating the cGMP-dependent kinase (PKG) can also stimulate 26S proteasomes and protein degradation, but in a manner distinct from PKA, and that these treatments also have beneficial effects in models of neurodegenerative diseases caused by an accumulation of mutant, misfolded proteins.

cGMP is an intracellular second messenger that is synthesized by soluble guanylyl cyclases in response to nitric oxide (17) or by transmembrane guanylyl cyclases in response to peptide hormones, such as natriuretic peptides (18). Most interest in cGMP has focused on its role in mediating smooth muscle relaxation and vasodilation, and many agents that raise cGMP are used clinically because of their beneficial effects on cardiovascular function. Inhibitors of phosphodiesterase 5 (PDE5; e.g., sildenafil or tadalafil), which raise cGMP by blocking its hydrolysis to GMP, are widely used for the treatment of erectile disfunction and pulmonary hypertension (19), and stimulators of soluble guanylyl cyclases (e.g., vericiguat or riociguat), which stimulate cGMP synthesis, are used as treatments for pulmonary hypertension and heart failure (17).

Although the ability of these agents to alter protein turnover generally or to combat the progression of neurodegenerative disease has received little or no attention, in a mouse model of a cardiomyopathy caused by overexpression of mutant αβ crystallin, raising cGMP with sildenafil was shown to reduce cardiac hypertrophy (10). This treatment also raised proteasomal peptidase activity in the heart and reduced the levels of mutant αβ-crystallin (10). These findings suggested that agents that raise cGMP may have therapeutic potential in hereditary cardiomyopathies. However, it was not clear whether these effects of cGMP on proteasomes are specific to the heart, whether PKG directly activates proteasomes, or whether cGMP may enhance intracellular protein breakdown generally and may also influence protein ubiquitination or autophagy.

Here we have systematically investigated whether different pharmacological agents that raise cGMP influence 26S proteasome function and the degradation of different classes of cell proteins. These studies demonstrate that PKG stimulates multiple 26S proteasome activities by phosphorylation of a different proteasome component than is modified by PKA. Also, unlike cAMP, cGMP stimulates the degradation of the bulk of cell proteins, and not just short-lived proteins. Because of these large increases in protein degradation, we also studied whether raising cGMP increases protein ubiquitination and autophagy. A number of major neurodegenerative diseases are caused by the accumulation of misfolded or mutant proteins, and in many cases the build-up of such aggregation-prone proteins impairs proteasome function, which leads to further defects in protein homeostasis (14, 20, 21). Because of the therapeutic potential of stimulating proteasome activity and enhancing the breakdown of disease-associated proteins, we also tested in zebrafish larvae models of tauopathies and Huntington’s disease whether raising cGMP or cAMP can stimulate the degradation of the causative mutant proteins and alleviate the associated pathologies.

## Results

### Pharmacological Agents That Raise cGMP Stimulate 26S Proteasome Activities.

**Fig. 1.**
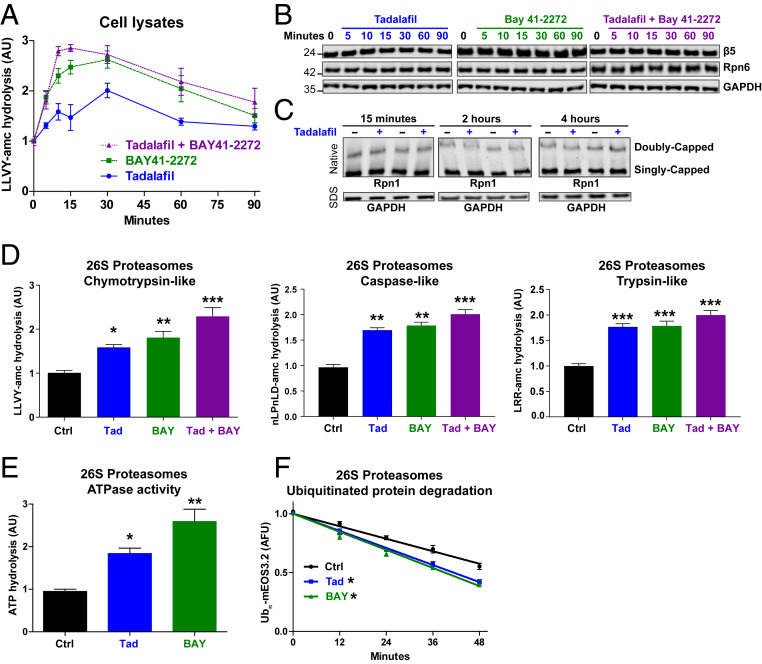
Pharmacological agents that raise cGMP stimulate 26S proteasome activities without changing proteasome amount. (*A*) Treatment of human neuroblastoma cells (SH-SY5Y) with the PDE5 inhibitor tadalafil (100 nM), the soluble guanylyl cyclase stimulator BAY41-2272 (100 nM), or a combination of the two increased proteasomal chymotrypsin-like activity in cell lysates. The linear rate of Suc-LLVY-AMC hydrolysis was used here and below as the measure of activity. In this and subsequent figures, error bars represent the means ± SEM. *n* = 4. (*B*) Levels of the 20S proteasome subunit β5 or the 19S subunit Rpn6 were not changed by treatments with pharmacological agents that raise cGMP. SH-SY5Y cells were treated with tadalafil (100 nM), BAY41-2272 (100 nM), or both, and the levels of proteasome subunits were analyzed by western blot. Representative western blots for one of three experiments is shown. (*C*) The amount of assembled 26S proteasomes was not changed by exposure of SH-SY5Y cells to the PDE5 inhibitor tadalafil. Cell lysates were analyzed by native PAGE and western blot with an antibody against the 19S subunit Rpn1. The same samples were also analyzed by sodium dodecyl sulfate polyacrylamide gel electrophoresis and western blot with an antibody against GAPDH to evaluate protein loading. Representative western blots for one of four experiments is shown. (*D*) After treatment of SH-SY5Y cells with these same agents (100 nM) for 30 min to raise cGMP, 26S proteasomes were affinity purified by the UBL method and exhibited greater chymotrypsin-like, caspase-like, and trypsin-like activities than those from control cells. *n* = 3 proteasome purifications. One-way ANOVA with Dunnett multiple comparison test. *P ≤ 0.05, **P ≤ 0.01, ***P ≤ 0.001. (*E*) 26S proteasomes purified from SH-SY5Y cells treated to raise cGMP as in B hydrolyzed ATP more quickly than proteasomes from control (Ctrl) cells. *n* = 3 proteasome purifications. One-way ANOVA with Dunnett multiple comparison test. *P ≤ 0.05, **P ≤ 0.01. (*F*) 26S proteasomes purified from SH-SY5Y cells treated with tadalafil (Tad) or BAY41-2272 (Bay) as in B degraded the fluorescent protein mEOS3.2 conjugated to a single K48-linked polyubiquitin chain more rapidly than proteasomes from control cells. *n* = 3 proteasome purifications. One-way ANOVA with Dunnett multiple comparison test. *P ≤ 0.05.

To raise the intracellular levels of cGMP, we used two inhibitors of PDE5, tadalafil or sildenafil, and two stimulators of soluble guanylyl cyclases, BAY41-2272 or cinaciguat. Addition of tadalafil (100 nM) or BAY41-2272 (100 nM) to human neuroblastoma cells (SH-SY5Y) caused a rapid increase in the proteasomes’ chymotrypsin-like peptidase activity in cell lysates (Fig. 1*A*). This increase was evident by 5 min after the addition of tadalafil or BAY41-2272 and was maximal at 30 min (Fig. 1*A*). As expected, the addition of tadalafil and BAY41-2272 together caused a faster increase in activity, which was maximal 10 min after their addition (Fig. 1*A*). This activity decreased between 30 and 90 min after addition, although it remained above control levels at 90 min (Fig. 1*A*).

This rapid stimulation and slow decline of peptidase activity occurred without any change in the levels of proteasome subunits (Fig. 1*B*). In addition, this cGMP-mediated stimulation of proteasome activity was not due to an increase in the assembly of the 26S proteasomes. When SH-SY5Y cells were treated with tadalafil, no change in the amounts of singly or doubly capped 26S proteasomes was seen at 15 min or 2 or 4 h later, as measured by native polyacrylamide gel electrophoresis (PAGE) and western blot (Fig. 1*C*).

To verify that these increases in peptide hydrolysis were due to greater 26S proteasome activity, SH-SY5Y cells were treated for 30 min with tadalafil, BAY41-2272, or these two agents together, and the 26S proteasomes were then purified using the UBL domain as the affinity ligand (22). The proteasomes from cells treated with tadalafil or BAY41-2272 exhibited 60 to 80% greater chymotrypsin-like activity, and the particles from cells incubated with both agents were approximately twice as active as those from control cells (Fig. 1*D*). Similar increases were also seen in the 26S proteasome’s caspase-like and trypsin-like activities (Fig. 1*D*). This coordinated enhancement of all three peptidase activities strongly suggests that activation involves more rapid entry of peptides into the 20S core, as occurs in other types of proteasome activation (23), rather than a stimulation of individual active sites.

The physiological substrates of 26S proteasomes are ubiquitinated proteins, whose degradation is linked to ATP hydrolysis (1). The proteasomes isolated from tadalafil or BAY41-2272–treated cells showed a greater capacity to hydrolyze ATP (Fig. 1*E*), and a polyubiquitinated protein, as assayed using as the substrate the fluorescent protein mEOS3.2 bearing a single K48-linked polyubiquitinated chain (24) (Fig. 1*F*). Thus, raising cGMP increases multiple activities of 26S proteasomes by a postsynthetic modification without increasing 26S proteasome assembly.

### PKG Activates Cytosolic, but Not Nuclear, Proteasomes.

**Fig. 2.**
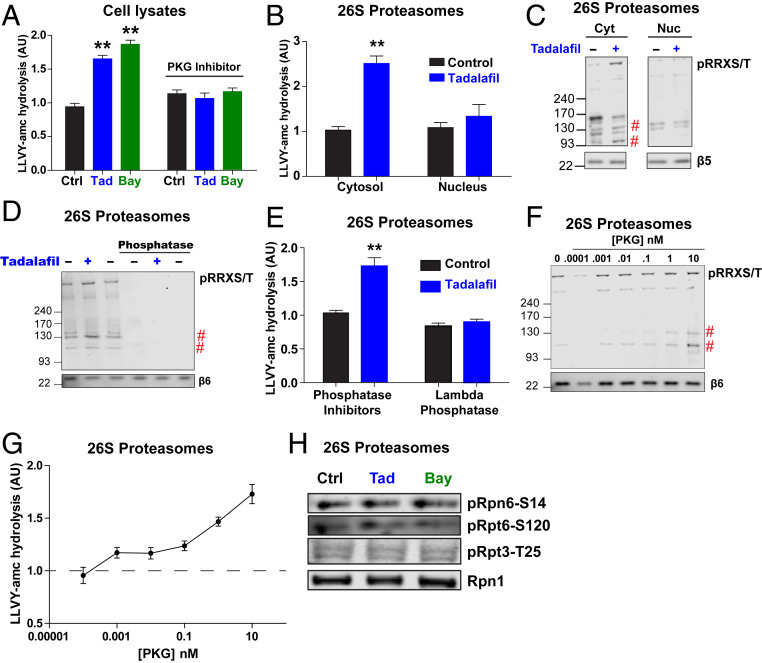
PKG directly activates 26S proteasomes by phosphorylation. (*A*) Inhibition of PKG with Rp-8-pCPT-cGMPs (1 μM) prevented the increase in proteasomal chymotrypsin-like activity caused by 30-min treatment of SH-SY5Y cells with tadalafil or BAY41-2272. *n* = 4 samples. One-way ANOVA with Dunnett multiple comparison test. **P ≤ 0.01. The experiment was repeated twice with similar results. (*B*) 26S proteasomes purified from the cytosolic fraction of tadalafil-treated SH-SY5Y cells showed increased chymotrypsin-like activity, but no increase was seen in the activity of 26S proteasomes from the nuclear fraction. *n* = 3 proteasome purifications. Student’s *t* test. **P ≤ 0.01. (*C*) Proteasomes purified from the cytosolic fraction of cells treated for 30 min with tadalafil contained increased amounts of phosphorylated proteins. 26S proteasome preparations from B were analyzed by western blot for phosphorylated RRXSer/Thr. The indicated bands (#) were reproducibly more intense in experiments from all proteasome preparation. Representative western blots from one of three proteasome purifications are shown. No phosphorylated RRXS/T bands were detected below 93 kDa. (*D*) Incubation of 26S proteasomes purified from SH-SY5Y cells with λ phosphatase for 1 h eliminated all bands containing phosphorylated Ser/Thr residues assayed as in B. Representative western blots from one of three experiments are shown. (*E*) Dephosphorylation of 26S proteasomes by λ phosphatase (shown in D) reversed the increase in chymotrypsin-like activity induced by treatment of SH-SY5Y cells with tadalafil for 30 min. *n* = 3 proteasome purifications. Student’s *t* test. **P ≤ 0.01. (*F*) Incubation of 26S proteasomes (5 nM) purified from human BJ5A fibroblast cells with increasing concentrations of PKG1α for 1 h caused an increase in the phosphorylation of the indicated bands (#). Representative western blots from one of two experiments is shown. (*G*) After proteasomes were incubated with increasing concentrations of PKG (F), their chymotrypsin-like activity increased in a progressive manner. The average activity ± SEM of three reactions per time point is shown. (*H*) Agents that raise cGMP do not cause the phosphorylation of the three sites on the proteasome previously reported to stimulate activity: Rpn6-S14 (13, 15), Rpt3-Thr25 (11), and Rpt6-S120 (27). 26S proteasomes purified from SH-SY5Y cells treated for 30 min with tadalafil or BAY41-2272 were analyzed by western blot for antibodies specific for the indicated phosphorylated subunits. Representative western blots from one of four proteasome purifications are shown.

Inhibiting the activation of PKG by treating SH-SY5Y cells with the cGMP analog Rp-8-Br-PET-cGMPs blocked the increases in the chymotrypsin-like activity induced by tadalafil and BAY41-2272 (Fig. 2*A*). Furthermore, transient overexpression of PKG1α in HEK293 cells stimulated this activity (*SI Appendix*, Fig. S1*A*). Although 26S proteasomes are abundant in both the nucleus and cytosol, PKG is localized primarily to the cytosol (25) (*SI Appendix*, Fig. S1*B*). Therefore, we tested whether activated PKG in cells stimulates the activities of proteasomes in both fractions. After treatment of SH-SY5Y cells with tadalafil or dimethyl sulfoxide (DMSO) for 30 min and hypotonic lysis, nuclei were separated from the cytosol by differential centrifugation and then 26S proteasomes were purified from each fraction by the UBL method. After tadalafil treatment, the cytosolic, but not the nuclear proteasomes, exhibited greater peptidase activity (Fig. 2*B*). Higher levels of protein phosphorylation were detected by western blot for pSer/Thr residues that are preceded by the amino acid sequence RRX (Fig. 2*C*). RRXpS/T is a consensus motif of PKG (26). Two phosphorylated bands of ∼130 kDa and 100 kDa were more intense across all conditions that raised cGMP and thus correlated with the increased proteasome activities. Because no proteasome subunit is larger than 100 kDa, the 130-kDa band must be a proteasome-associated protein.

### Phosphorylation Is Required for the Proteasome Activation Caused by Intracellular cGMP and PKG Phosphorylates and Activates Purified Proteasomes.

To verify that phosphorylation of proteasome subunits or a proteasome-associated protein is responsible for the activation by cGMP and PKG, 26S proteasomes were affinity-purified from SH-SY5Y cells after a 30-min treatment with tadalafil or DMSO and incubated with λ phosphatase. This enzyme reduced the levels of phosphorylated proteins (Fig. 2*D*) and eliminated the tadalafil-mediated increase in chymotrypsin-like activity (Fig. 2*E*).

Because PKG may activate proteasomes indirectly in cells by phosphorylating another kinase or a cytosolic protein, which then might bind and activate the proteasomes, we tested whether PKG can phosphorylate and activate the affinity-purified 26S proteasomes. After incubation of 26S proteasomes from human fibroblasts (BJ5A) with increasing concentrations of human PKG1α for 1 h, the levels of phosphorylated bands at 100 and 130 kDa in the proteasome preparations increased in a concentration-dependent manner (Fig. 2*F*). There was also a concentration-dependent increase in the proteasome’s peptidase activity (Fig. 2*G*). Thus, phosphorylation is essential for proteasome activation, and PKG in vitro can stimulate 26S activity by phosphorylating a proteasome subunit or a tightly associated regulatory protein.

### PKG Does Not Phosphorylate Rpn6, Rpt3, or Rpt6.

Prior work has demonstrated that phosphorylation of two sites on the 19S regulatory particle increases proteasomal activities: Rpn6-S14 by PKA (13, 15) and Rpt3-Thr25 by DYRK2 (11, 12). Using phosphosite-specific antibodies, we tested if levels of these phosphorylated subunits increase in 26S proteasomes purified from tadalafil- or BAY-412272–treated SH-SY5Y cells. Neither treatment increased the phosphorylation of these sites (Fig. 2*H*), nor did transient overexpression of PKG1α in HEK293 cells (*SI Appendix*, Fig. S1*C*). However, the overexpression of PKG did increase the amount of phosphorylated proteins detected in the proteasome preparations above levels in particles from cells transfected with an empty vector. In addition, the overexpressed PKG copurified with the 26S proteasomes (*SI Appendix*, Fig. S1*C*). Similar increases in the levels of phosphorylated proteins and the amount of PKG that copurified with proteasomes were seen after treatment of HEK293 cells with the soluble guanylyl cyclase stimulator cinaciguat (*SI Appendix*, Fig. S1*D*).

The subunit Rpt6 has also been reported to be phosphorylated by PKG (10) and on S120 by CAMKII (3). However, using an antibody specific for phosphorylated Rpt6-S120 (27), we found no change in phosphorylation of this site in proteasomes purified from tadalafil- or BAY41-2272–treated SH-SY5Y cells (Fig. 2*H*). Thus, PKG directly stimulates proteasomal activity by phosphorylation of a 26S subunit or a protein that copurifies with the 26S particle, that is not presently known to regulate proteasome function.

### Raising cGMP Stimulates Degradation of Both Short-Lived and Long-Lived Cell Proteins.

**Fig. 3.**
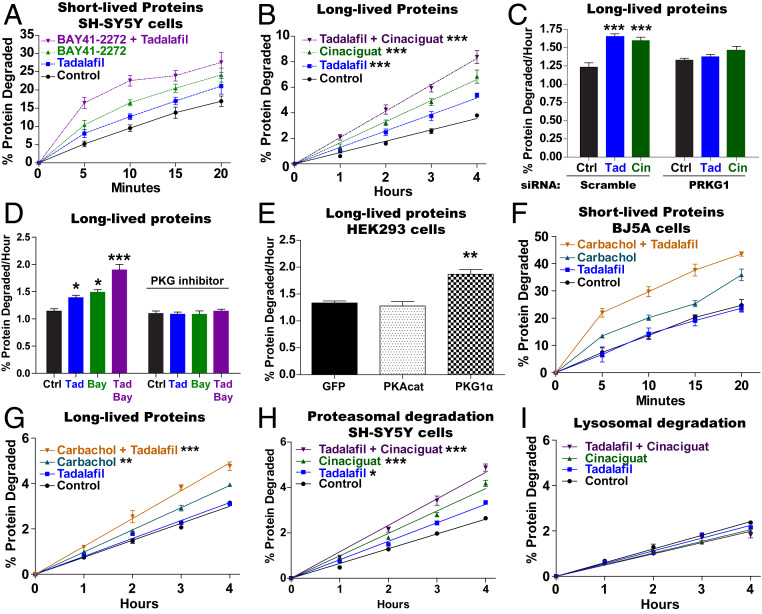
Raising cGMP stimulates total intracellular protein breakdown by the UPS. (*A*) Raising cGMP stimulates the degradation of short-lived intracellular proteins. SH-SY5Y cells were incubated for 20 min with ^3^H-phenylalanine, and then protein degradation was measured in the presence of cycloheximide (150 µg/mL) by following the conversion of ^3^H-labeled proteins to amino acids in the media. The pharmacological agents were added to the media after the labeling of cell proteins. *n* = 4. Error bars represent the means ± SEM. (*B*) The degradation of long-lived proteins, the bulk of cell proteins, was increased by agents that raise cGMP. SH-SY5Y cells were exposed to ^3^H-phenylalanine for 16 h and then washed and resuspended in chase medium containing excess nonlabeled phenylalanine for 2 h. The conversion of radiolabeled proteins to labeled amino acids in the media was measured. The pharmacological agents were added to the media after the 2-h chase period. Data shown are the slopes calculated from linear degradation rates. *n* = 3. One-way ANOVA with Dunnett multiple comparison test. ***P ≤ 0.001. (*C*) Knockdown of PKG with siRNA blocked the cGMP-mediated increase in the degradation of long-lived proteins. SH-SY5Y cells were exposed to scramble- or PRKG1-siRNA for 48 h, and then degradation of long-lived proteins was measured as in B. A representative western blot of one of three knockdowns of PKG is shown in *SI Appendix*, Fig. S4*C*. *n* = 3. One-way ANOVA with Dunnett multiple comparison test. ***P ≤ 0.001. (*D*) Inhibition of PKG with Rp-8-pCPT-cGMPs (1 μM) blocked the increase in the degradation of long-lived proteins caused by agents that raise cGMP. Protein degradation was measured as in B. *n* = 3. One-way ANOVA with Dunnett multiple comparison test. *P ≤ 0.05, ***P ≤ 0.001. (*E*) Overexpression of PKG, but not PKA catalytic subunit, stimulated the degradation of long-lived proteins. HEK293 cells were transfected with cDNA encoding GFP, PKA catalytic subunit, or PKG 6 h before exposure to ^3^H-phenylalanine. Protein degradation was measured as in *B*. *n* = 3. One-way ANOVA with Dunnett multiple comparison test. **P ≤ 0.01. (*F*) The muscarinic agonist carbachol (1 µM) increases the degradation of short-lived proteins in human fibroblasts (BJ5A) and increases degradation even further in the presence of the PDE5 inhibitor tadalafil. Protein degradation was measured as in A, and all agents were added after the labeling of cell proteins with ^3^H-phenylalanine. *n* = 4. (*G*) Carbachol also increases the degradation of long-lived proteins in human fibroblasts (BJ5A) and increases degradation even further in the presence of the PDE5 inhibitor tadalafil. Protein degradation was measured as in *B*. *n* = 3. One-way ANOVA with Dunnett multiple comparison test. **P ≤ 0.01, ***P ≤ 0.001. (*H*) Degradation of long-lived cell proteins by the proteasome was increased by agents that raise cGMP. Protein degradation was measured as in *B*, but the contribution of the proteasome was measured by including in the media the inhibitor of lysosomal acidification concanamycin A (200 nM) 1 h after protein labeling. *n* = 4. One-way ANOVA with Dunnett multiple comparison test. *P ≤ 0.05, ***P ≤ 0.001. (*I*) Lysosomal protein degradation (autophagy) was not increased by treatments that raise cGMP. The degradation of long-lived proteins was measured as in *B*, and to measure the contribution of the lysosome, the proteasome inhibitor carfilzomib (1 µM) was added to the media in the second hour of the chase period and was present throughout the measurements of degradation. *n* = 4.

Raising the level in cells of cAMP with pharmacological agents or hormones enhances selectively the degradation of short-lived proteins (13, 15), which include misfolded, damaged, and regulatory proteins, but does not alter the breakdown of long-lived proteins, which comprise the bulk of cell components. To determine whether cGMP and PKG also stimulate the degradation of short-lived proteins, SH-SY5Y cells were incubated with ^3^H-phenylalanine for 20 min to label short-lived proteins. After the ^3^H-phenylalanine was removed, fresh media containing large amounts of nonradioactive phenylalanine and cycloheximide were added to prevent reincorporation of the labeled amino acids released by proteolysis. The degradation of the labeled proteins was then measured by assaying the generation of radiolabeled amino acids (28). Adding tadalafil or BAY41-2272 after the incorporation of ^3^H-phenylalanine increased the rate of degradation of the most short-lived proteins, and combining these two agents led to an even greater increase in their degradation (Fig. 3*A*). A similar increase in degradation of short-lived proteins was seen in SH-SY5Y cells with tadalafil, cinaciguat, and a combination of these two agents (*SI Appendix*, Fig. S2).

To determine whether raising cGMP also stimulates the degradation of damaged or misfolded proteins, which are short-lived, we induced the production of incomplete proteins by exposing SH-SY5Y cells for 1 h to puromycin, which is incorporated into newly synthesized proteins and causes premature termination of the polypeptides which are then rapidly hydrolyzed (29). Degradation of the puromycyl polypeptides was followed in the presence of cycloheximide by western blot for puromycin. Adding tadalafil with the cycloheximide reduced the amount of puromycin-containing polypeptides below the levels in control cells (*SI Appendix*, Fig. S3*A*). Thus, cGMP and PKG enhance the degradation of these abnormal polypeptides.

These increases in proteolysis presumably also reflect accelerated degradation of many short-lived regulatory proteins. Therefore, we measured by western blot the degradation in the presence of cycloheximide of p53, whose rapid ubiquitination and degradation have been extensively characterized (30). The breakdown of p53 was also increased by treatment with tadalafil or BAY41-2272 (*SI Appendix*, Fig. S3*B*).

To evaluate whether raising cGMP also increases the degradation of the bulk of cell proteins, which are long-lived components, SH-SY5Y cells were incubated with ^3^H-phenylalanine for 16 h, chased for 2 h with nonradiolabeled phenylalanine to allow the hydrolysis of labeled short-lived proteins, and then the degradation of the ^3^H-labeled long-lived proteins was measured (31). Addition of tadalafil or cinaciguat after the 2-h chase increased the rate of degradation of long-lived proteins by 30 to 50%, and a combination of the two agents caused an even larger (approximately twofold) increase in the overall rate of proteolysis (Fig. 3*B*). A similar stimulation of proteolysis was seen in SH-SY5Y with sildenafil and BAY41-2272 and also in C2C12 mouse myotubes (*SI Appendix*, Fig. S4 *A* and *B*). These rates of degradation of long-lived proteins were linear for at least 4 h and were surprisingly high. In fact, these rates were greater than those seen in such cells upon serum or nutrient deprivation (31), and the twofold increase with tadalafil and cinaciguat together exceeds rates of degradation seen in highly catabolic conditions (e.g., muscle atrophy or cachexia) (32). Thus, raising cGMP, like raising cAMP, stimulates the degradation of short-lived proteins, but unlike cAMP, it also increases degradation of the long-lived fraction.

The cGMP-mediated increase in protein degradation required PKG because it could be blocked in SH-SY5Y cells either by knockdown of PKG1 with small interfering RNA (siRNA) (Fig. 3*C* and *SI Appendix*, Fig. S4*C*) or by inhibition of PKG with Rp-8-Br-PET-cGMPs (Fig. 3*D*). Furthermore, the overexpression in HEK293 cells of PKG1α increased the degradation of long-lived proteins (Fig. 3*E*). In contrast, overexpression of PKA’s catalytic subunit did not enhance the degradation of those long-lived proteins, in accord with our prior findings (13, 15). Thus, increasing PKG activity is necessary and sufficient for the cGMP-mediated increase in protein degradation.

The neurotransmitter, acetylcholine, raises levels of cGMP via M2 muscarinic receptors and treating cultured rat cardiomyocytes with M2 agonists was reported to stimulate proteasomal peptidase activity through PKG (9). To test whether raising cGMP via M2 muscarinic receptors also stimulates total protein breakdown in cells, we used the immortalized human foreskin fibroblast cell line, BJ5A-hTERT, which expresses M2 muscarinic receptors but not other muscarinic or nicotinic receptors (humanproteinatlas.org). In these cells, as in other lines tested, cinaciguat stimulated the degradation of both short- and long-lived proteins (*SI Appendix*, Fig. S5 *A* and *B*). Surprisingly, tadalafil by itself did not increase protein degradation in these cells (*SI Appendix*, Fig. S5 *A* and *B*) or in HEK293 cells (*SI Appendix*, Fig. S5 *C* and *D*), probably due to a low level of basal cGMP synthesis. However, combining cinaciguat with tadalafil in BJ5A and HEK293 cells produced even larger increases in proteolysis (*SI Appendix*, Fig. S5). Treating BJ5A cells with carbachol, a pan muscarinic receptor agonist, stimulated the degradation of both short- and long-lived proteins (Fig. 3 *F* and *G*), and these rates could be further increased if tadalafil was also present (Fig. 3 *F* and *G*). These observations with carbachol suggest that an increase in degradation occurs in vivo when cGMP is raised by cholinergic stimuli.

### Raising cGMP Stimulates Proteasomal Degradation but Not Autophagy.

Long-lived cell proteins are degraded primarily by the UPS, but also by autophagy (31). To determine the contribution of each pathway to the cGMP-mediated increase in overall protein breakdown, a specific irreversible inhibitor of the proteasome, carfilzomib, was included in the chase period to measure degradation by lysosomes, or an inhibitor of lysosomal acidification, concanamycin A, to measure proteasomal degradation (28). Tadalafil and cinaciguat alone or together stimulated protein degradation to a similar extent in the presence of concanamycin A, and thus they clearly promote proteasomal proteolysis (Fig. 3*H*). In contrast, neither agent, alone or in combination, increased protein degradation when proteasomes were inhibited (Fig. 3*I*). Thus, the stimulation of overall protein breakdown by PKG occurs through a selective increase in proteolysis by the proteasomes.

### cGMP Increases Levels of Ubiquitinated Proteins by Stimulating Ubiquitin Conjugation.

**Fig. 4.**
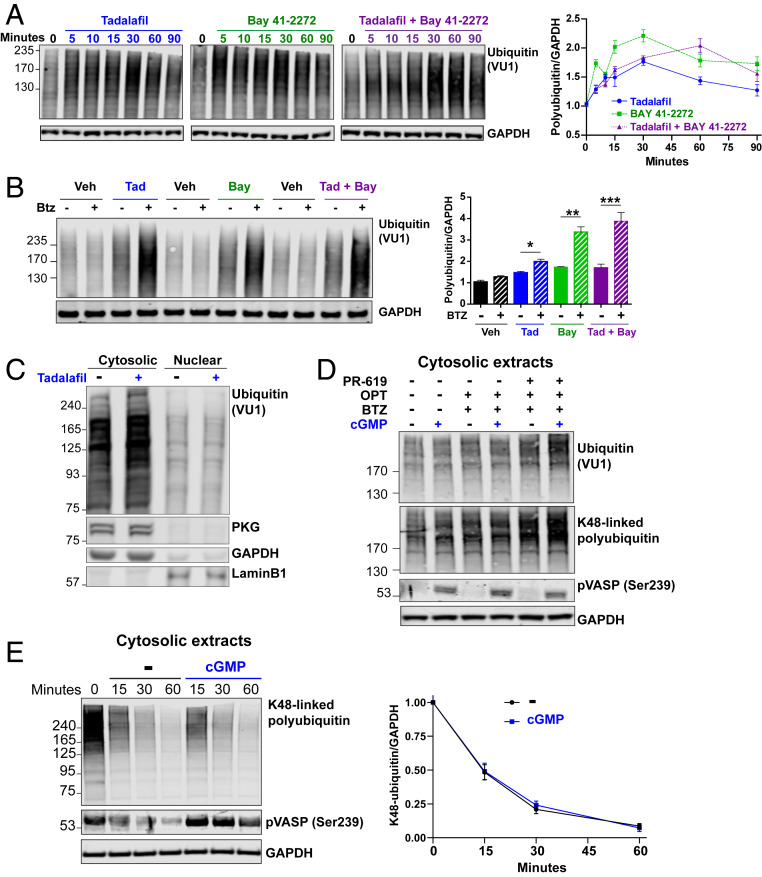
cGMP also rapidly increases levels of ubiquitin conjugates by stimulating ubiquitylation of cell proteins. (*A*) SH-SY5Y cells were treated with agents that raise cGMP and the cell lysates were analyzed by western blot for ubiquitin. The levels of polyubiquitinated proteins were quantified and representative western blots from one of three experiments are shown. *n* = 3. Averages ± SEM are shown. (*B*) The increase in ubiquitin conjugates caused by agents that raise cGMP is even greater in the presence of the proteasome inhibitor bortezomib. SH-SY5Y cells were treated for 15 min with these agents, lysed, and analyzed by western blot for ubiquitin, as in *A*. Representative western blots of one of four experiments are shown. *n* = 4. One-way ANOVA with Bonferroni’s multiple comparison test. *P ≤ 0.05, **P ≤ 0.01, ***P ≤ 0.001. (*C*) Raising cGMP with tadalafil for 30 min in SH-SY5Y cells increases levels of polyubiquitinated proteins in the cytosolic fraction, but not the nuclear fraction. Representative western blots of one of three experiments are shown. (*D*) cGMP increases ubiquitination of cytosolic proteins in a cell-free system. HEK293 cells were lysed in a hypotonic buffer, and then cytosolic extracts were prepared by pelleting the nucleus (800 × *g* for 15 min) and mitochondria (10,000 × *g* for 10 min). The extracts were incubated for 30 min at 37 °C with or without cGMP (1 µM) in the presence of bortezomib (1 µM), 1,10-phenanthroline (250 µM), and PR-619 (10 µM) to prevent deubiquitination and proteasomal degradation of ubiquitin conjugates. Representative western blots of one of four experiments is shown. (*E*) cGMP does not change the rate of deubiquitination of cytosolic proteins in a cell-free system. HEK293 cells were treated for 30 min with bortezomib and then cytosolic extracts were prepared as in *D*, except an inhibitor of the ubiquitin activating enzyme (TAK243, 1 µM) was included instead of inhibitors of deubiquitinases. Representative western blot is shown of one of three experiments. *n* = 3.

Because raising cGMP causes such large increases in proteasomal degradation, we also tested whether these treatments, in addition to stimulating proteasome activities, might enhance the ubiquitination of cell proteins. Surprisingly, adding tadalafil or BAY41-2272 to SH-SY5Y cells increased within 5 min the total cellular content of ubiquitinated proteins (Fig. 4*A*). This increase in ubiquitinated proteins was maximal 30 min after exposure to either agent and then decreased during the subsequent 60 min, although the level of ubiquitin conjugates still remained higher than in control cells (Fig. 4*A*). Combining tadalafil and BAY41-2272 also raised the levels of ubiquitin conjugates within 5 min, but the maximal increase (twofold) was not reached until 60 min (Fig. 4*A*). Surprisingly, this accumulation of polyubiquitinated proteins with the combined treatment appeared slower than with either agent alone, perhaps because of the faster activation of proteasomes (Fig. 1*A*) and greater degradation of ubiquitinated proteins (Fig. 3). In fact, the changes shown in Fig. 4*A* must underestimate the actual increase in levels of ubiquitin conjugates due to the simultaneous enhancement of proteasomal degradation. Accordingly, when SH-SY5Y cells were treated with the agents that raise cGMP plus the proteasome inhibitor bortezomib for 15 min, the levels of ubiquitinated proteins increased even further (Fig. 4*B*). Tadalafil treatment for 30 min in SH-SY5Y cells also increased the levels of polyubiquitinated proteins only in the cytosolic fraction, where PKG is localized, and where proteasomes are activated (Fig. 2*B*), and not in the nuclear fraction (Fig. 4*C*).

To determine whether the increased levels of polyubiquitinated proteins were due to increased ubiquitination or decreased deubiquitination of cell proteins, we used cytosolic extracts from HEK293 cells from which nuclei and mitochondria were sequentially removed by differential centrifugation. Incubating these extracts with 1 µM cGMP for 30 min in the presence of the broad-spectrum phosphodiesterase inhibitor IBMX activated PKG, as shown by increased phosphorylation of vasodilator-stimulated phosphoprotein (VASP) (Fig. 4*D* and *SI Appendix*, Fig. S6*A*), a well-characterized PKG substrate, and increased proteasomal peptidase activity (*SI Appendix*, Fig. S6*B*), as also seen upon treatment of cells with agents that raise cGMP (Fig. 1) and incubation of purified 26S particles with PKG (Fig. 2*G*). Addition of cGMP to the extracts in the presence of bortezomib to inhibit proteasomes, 1,10-phenanthroline to inhibit metallo-deubiquitinases and PR-619 to inhibit a broad spectrum of deubiquitinases (33), increased the levels of polyubiquitinated proteins and K48-linked polyubiquitinated proteins, strongly suggesting a stimulation of protein ubiquitination (Fig. 4*D*). In contrast, cGMP did not change the levels of K63-linked or K11-linked polyubiquitinated proteins (*SI Appendix*, Fig. S7).

To evaluate whether cGMP also decreases the rate of deubiquitination of cell proteins, HEK293 cells were treated for 30 min with bortezomib to increase the levels of ubiquitinated proteins and cytosolic extracts were prepared as above, but in the presence of an inhibitor of the ubiquitin-activating enzyme instead of the inhibitors of deubiquitinases. Adding cGMP to these extracts did not change the rate of deubiquitination of cell proteins (Fig. 4*E*). Furthermore, cell lysates prepared from SH-SY5Y cells treated for 30 min with tadalafil, cinaciguat, or both, did not exhibit greater deubiquitinating activity than control, as measured by the covalent modification of the active sites of deubiquitinating enzymes with HA-Ub-VS (*SI Appendix*, Fig. S8*A*) and the hydrolysis of ubiquitin-amc (*SI Appendix*, Fig. S8*B*). Thus, cGMP increases the levels of polyubiquitinated proteins by stimulating the ubiquitination of cell proteins more than it enhances their degradation by proteasomes.

### Raising cGMP or cAMP Reduces Pathology in Zebrafish Models of Neurodegenerative Diseases.

**Fig. 5.**
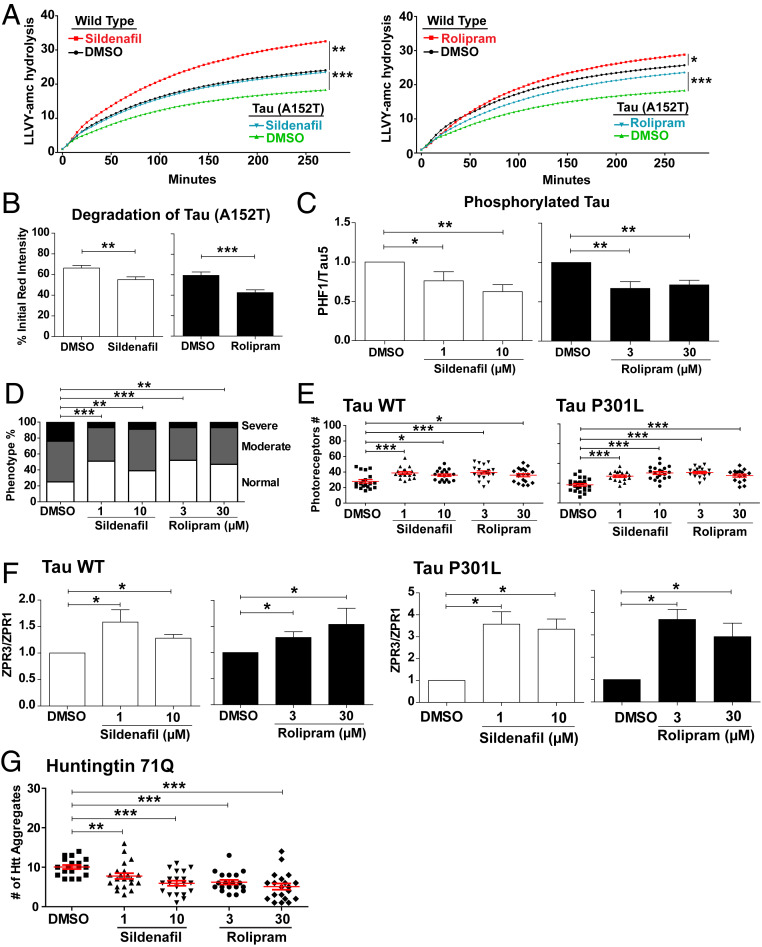
Sildenafil and rolipram treatment stimulate proteasome activity and reduce the levels of toxic proteins, cell death, and morphological abnormalities in zebrafish models of neurodegenerative diseases. (*A*) Addition of sildenafil to raise cGMP or rolipram to raise cAMP increased proteasome activity in WT fish and in fish overexpressing Dendra-tau-A152T. Proteasomal peptidase activity in fish overexpressing Dendra-tau-A152T was less than in control fish (35). The proteasomes’ chymotrypsin-like activity in fish lysates was measured using Suc-LLVY-AMC (*n* = 15 per group) after a 1-d treatment. Data represent mean values ± SEM here and below. Two-way ANOVA test. *P ≤ 0.05, **P ≤ 0.01, ***P ≤ 0.001. (*B*) Sildenafil and rolipram treatment for 6 h increased the rate of tau clearance (*n* = 50 neurons per group). In vivo tau clearance of photoconverted “red” Dendra-tau was measured within individual neurons in the spinal cord. The measurement of the intensity of the photoconverted Dendra-tau signal at 6 h relative to initial red intensity reflects the clearance of tau protein. Two-tailed unpaired *t* test. **P ≤ 0.01, ***P ≤ 0.001. (*C*) The levels of hyperphosphorylated tau relative to total tau in Dendra-tau-A152T fish decreased after treatment with sildenafil or rolipram (*n* = 10 per group). The accumulation of hyperphosphorylated tau is one of the hallmarks of tauopathies. Representative western blots shown in *SI Appendix*, Fig. S10*A*. One-way ANOVA with post hoc Tukey’s multiple comparison test. *P ≤ 0.05, **P ≤ 0.01. (*D*) Exposure to sildenafil and rolipram for 2 d ameliorates the morphological defects in Dendra-tau-A152T fish. Both treatments increased the percentage of larvae with normal phenotypes and reduced the proportion of deformed larvae (*n* = 100 per treatment group). χ^2^ test with confidence interval 95%. **P ≤ 0.01, ***P ≤ 0.001. (*E*) Quantification of photoreceptors from images of sections through the central retina (*n* ≥ 16 per group) showed that both sildenafil and rolipram increased the survival of photoreceptors above levels in siblings treated with DMSO. Representative images shown in *SI Appendix*, Fig. S10*B*. One-way ANOVA with post hoc Tukey’s multiple comparison test. *P ≤ 0.05, ***P ≤ 0.001. (*F*) Sildenafil or rolipram treatment reduce the degeneration of rod photoreceptors in Rho::EGFP-TauWT and Rho::EGFP-TauP301L zebrafish larvae. Densitometry of western blots for the major rod photoreceptor protein rhodopsin (ZPR3) relative to the loading control arrestin (ZPR1) from three independent experiments (*n* = 10 per group). Representative western blots used for quantification are shown in *SI Appendix*, Fig. S10*C*. One-way ANOVA with post hoc Tukey’s multiple comparison test. *P ≤ 0.05. (*G*) In Rho::EGFP-HD71Q transgenic fish, in which the expression of the mutant form of huntingtin exon 1 (71Q) leads to aggregate formation and degeneration of rod photoreceptors sildenafil and rolipram treatment decreased the number of huntingtin aggregates below the levels in the control (DMSO-treated) group. One-way ANOVA with post hoc Tukey’s multiple comparison test. **P ≤ 0.01, ***P ≤ 0.001.

Many neurodegenerative diseases are caused by an accumulation of aggregation-prone misfolded proteins, and there is growing evidence in experimental models of these disease that such proteins lead to impaired proteasome activity and a reduced cellular capacity for protein breakdown (14, 20). Agents that raise cAMP appear to be promising therapies for proteotoxic diseases through their ability to enhance proteasome activity and the degradation of such toxic proteins (13, 14, 16). Because cGMP can also activate proteasomes and enhance protein degradation, we tested whether raising cGMP might also help clear these aggregation-prone proteins and thus reduce the resulting pathology in zebrafish models of tauopathies and Huntington’s disease. To determine whether raising cGMP or cAMP can activate proteasomes in zebrafish, larvae were exposed to sildenafil (1 and 10 µM), rolipram (3 and 30 µM), or DMSO for 5 d. Proteasomes purified by the UBL method from larvae treated with either concentration of sildenafil or rolipram exhibited greater chymotrypsin-like activity than those from the DMSO-treated or untreated larvae (*SI Appendix*, Fig. S9*C*). Furthermore, exposure to these agents for only 1 d also increased proteasomal peptidase activity in the lysates of WT larvae (Fig. 5*A*).

At these concentrations, neither drug had any overt toxic effects on the larvae. There was also no change in the levels of proteasome subunits in the lysates of the larvae, and the treatments (*SI Appendix*, Fig. S9*A*) with 1 µM sildenafil or 3 µM rolipram did not alter the levels of assembled 26S proteasomes in the lysates as measured by native PAGE and western blot (*SI Appendix*, Fig. S9*B*). In these lysates, unlike those of human cells (Fig. 1*C*), the singly capped 26S proteasomes ran as a doublet on native PAGE (*SI Appendix*, Fig. S9*B*). Treatment with 10 µM sildenafil or 30 µM rolipram, but not with the lower concentrations of either agent, increased the intensity of the upper band of the doublet and decreased the intensity of the lower band (*SI Appendix*, Fig. S9*B*). This interesting effect, however, did not correlate with changes in activity because 26S proteasomes from larvae treated with 1 or 10 µM sildenafil or 3 or 30 µM rolipram showed similar increases in peptidase activity (*SI Appendix*, Fig. S9*C*).

Because raising cGMP and cAMP activate 26S proteasomes via postsynthetic modification in zebrafish, as in mammalian cells, we investigated their possible effects in models of neurodegenerative diseases. The A152T mutation in tau greatly increases the risk of developing frontotemporal dementia in humans (34), and expression of this mutant tau in the nervous system of zebrafish larvae causes neurodegeneration, an accumulation of hyperphosphorylated tau, as occurs in human tauopathies, and impairment of proteasomal peptidase activity in the lysates (35) (Fig. 5*A*). However, exposure to sildenafil or rolipram for 1 d stimulated proteasomal peptidase activity toward the levels in vehicle-treated WT larvae (Fig. 5*A*).

To determine whether these treatments also enhanced the degradation in vivo of the A152T mutant, the dendra-tag fused to the mutant tau was photoconverted from green to red, and then the larvae were treated with sildenafil or rolipram. The amount of red mutant tau was assayed in individual spinal cord neurons 6 h after photoconversion. Both sildenafil and rolipram increased the degradation of mutant tau (Fig. 5*B*). In addition, these treatments reduced the levels of hyperphosphorylated tau detected by western blot relative to the total amount of tau (Fig. 5*C*) (*SI Appendix*, Fig. S10*A*). Most of the zebrafish larvae expressing the A152T mutant tau in the nervous system exhibit an abnormal curvature of the dorsal spine (35), with 50% having spinal curvature that was classified as moderate and 25% as severe (Fig. 5*D*). Treatment with either sildenafil or rolipram for 2 d increased the percentage of the clutch with no curvature from 25 to ∼50% (Fig. 5*D*) and decreased the percentage of larvae with a severe curvature from 25 to ∼10% (Fig. 5*D*). Thus, both sildenafil and rolipram not only restored proteasomal activity, but also reduced the levels of pathological tau and the associated morphological defects caused by A152T tau.

Overexpression in the zebrafish retina, under the rhodopsin promoter, of human WT tau or the P301L mutant tau, which also causes frontotemporal dementia, leads to degeneration and death of the rod photoreceptors (36). To determine if raising cGMP or cAMP could prevent this cell death, fish larvae overexpressing either Rho::EGFPTauWT or Rho::EGFPTauP301L were exposed to sildenafil or rolipram for 5 d and the photoreceptors detected in the central retina were counted. Both treatments increased the number of rod photoreceptors (Fig. 5*E* and *SI Appendix*, Fig. S10*B*). Furthermore, the degree of rod photoreceptor degeneration was determined by analyzing the ratio of the rod protein ZPR3 (rhodopsin) to the cone protein ZPR1 (arrestin) measured by western blot. Both sildenafil and rolipram increased this ratio compared to that seen in siblings that received only the vehicle (Fig. 5*F* and *SI Appendix*, Fig. S10*C*), indicating that these agents prevented the degeneration phenotype. This effect was not due to decreased expression of the transgene, because these treatments did not change the levels of EGFP mRNA measured by qRT-PCR (*SI Appendix*, Fig. S10*D*).

In Huntington’s disease, mutant huntingtin containing an expanded polyglutamine repeat accumulates in protein aggregates within neurons. To test whether raising cAMP or cGMP could also reduce the levels of such aggregates, zebrafish larvae expressing exon 1 of human huntingtin with 71 glutamine repeats in rod photoreceptor cells (Rho::EGFP-HD71Q) were treated for 5 d with sildenafil or rolipram, and the number of microscopically visible aggregates in the retina was measured. Both agents reduced the number of huntingtin aggregates (Fig. 5*G*). Thus, these agents can activate proteasomes, enhance the clearance of aggregation-prone proteins, and reduce the resulting neuronal death and morphological abnormalities.

## Discussion

### A Mechanism for Regulating Overall Protein Breakdown in Cells.

The regulation of protein breakdown by cGMP and PKG differs in several respects from other known mechanisms that control intracellular proteolysis. Pharmacological agents that inhibit PDE5 or stimulate soluble guanylate cyclase rapidly increase not only the activities of 26S proteasomes, but also the levels of ubiquitinated proteins and the total rate of intracellular protein degradation. Raising cGMP content even further by combining these treatments caused a further increase in the magnitude, rapidity, and duration of these responses. The increase in ubiquitin conjugates, proteasomal degradation, and overall proteolysis were all evident within 5 min after addition of these drugs to cultured cells and these changes occurred too rapidly to result from changes in gene transcription. It is noteworthy that these increases in proteasomal activity and ubiquitinated proteins were only observed in the cytosol, where PKG is localized, not the nucleus. The stimulation of proteasome activity and protein turnover by cGMP and PKG appears to be a general cellular response because similar changes were seen in HEK293, neuroblastoma, myotubes, and fibroblasts, as well as zebrafish larvae. However, cell types differ widely in their content of phosphodiesterases and guanylate cyclases and in the physiological factors that promote cGMP synthesis. Therefore, it remains to be determined to what extent in different tissues, hormones (e.g., natriuretic peptides), signaling molecules (e.g., NO), neurotransmitters (e.g., acetylcholine), or drugs that raise cGMP also stimulate proteasome function and overall proteolysis by the UPS.

Although both cGMP and cAMP enhance proteasome activity and the degradation of short-lived proteins, they probably do not stimulate the ubiquitination and degradation of the same short-lived proteins. cGMP and cAMP are generated in different physiological states and often have opposite effects on cell function, especially in the cardiovascular system. In hearts, epinephrine via cAMP increases cardiac output, while acetylcholine via M2 muscarinic receptors raises cGMP and decreases cardiac output. PKG’s ability to also stimulate the breakdown of the bulk of cell proteins clearly distinguishes its effects on protein degradation from those of PKA and must have important physiological consequences. For example, in hearts adrenergic agonists via cAMP induce hypertrophy (37), while agents that raise cGMP reduce this pathologic process (38). The capacity of cGMP to increase the overall breakdown of cell proteins may contribute to this inhibition of hypertrophy.

The increases in overall degradation by the UPS with these pharmacological treatments were surprisingly large and even exceeded the increases in proteolysis reported in cells in various catabolic states, such as cachexia and muscle atrophy (29) or upon heat shock (37). The approximately twofold increase in total proteolysis induced by the combination of sildenafil and cinaciguat is also much larger than the 40 to 60% increases seen typically in cultured cells upon serum deprivation (31) or mammalian target of rapamycin (mTOR) inhibition (28), both of which stimulate proteolysis primarily by increasing autophagy with only about a 20% enhancement in proteasomal degradation (28). Such a large cGMP-induced increase in total protein degradation, if maintained, should by itself lead to a clear decrease in cell mass within a few days, depending on the cell’s rate of protein synthesis. However, a net loss of cell proteins may not develop if rates of protein synthesis are also stimulated by cGMP, or if the effects on proteasomes and protein degradation are not maintained. Clearly, our findings raise the possibility that in certain catabolic states or during cellular transitions, cGMP may signal a global increase in proteolysis and thus influence cell size and composition.

### Activation of Proteasomal Function through Phosphorylation by PKG.

A variety of protein kinases have been reported to alter proteasome activities (2), although only three—PKA (13, 15), DYRK2 (11), and now PKG—have been shown to activate 26S proteasomes by phosphorylation and to promote intracellular protein degradation. The findings that phosphatase treatment reverses proteasome activation by cGMP in cells and that incubation with pure PKG causes activation of purified proteasomes clearly demonstrate that the stimulation of proteasome activities involves direct phosphorylation by PKG of a 26S subunit or a 26S-associated regulatory protein. PKG must be phosphorylating a 26S subunit or an associated protein not currently known to regulate proteasome activities, because after activation by PKG in vitro or in cells, no change was seen with antibodies specific for the three phosphopeptides reported to increase proteasome activities: Rpn6-S14 (13, 15), Rpt3-Thr25 (11), or Rpt6-S120 (27). However, despite repeated attempts, we have been unable thus far to identify the proteasomal protein modified by PKG by various mass spectrometric approaches. For reasons that remain unclear, the identification of the critical phosphorylated sites on proteasomes has proven surprisingly difficult for us and for other investigators (5).

It has been claimed that increases in proteasome activity are associated with or even require greater formation of 26S proteasomes (39). However, raising cGMP levels by several approaches did not change the levels of proteasome subunits or the content of doubly or singly capped 26S particles. Thus, the increase in proteasome activity is not due to the synthesis or assembly of new proteasomes but through a postsynthetic modification of preexistent 26S particles.

Because these PKG-induced changes in proteasome activities resemble those induced by PKA, it is unclear (13, 15) how similar alterations in proteasome activity can lead to the distinct effects of PKG and PKA on proteolysis in cells (i.e., how both kinases can stimulate the degradation of short-lived fraction of cell proteins, but only PKG enhances the breakdown of long-lived components). Possibly, after phosphorylation by PKA or PKG, proteasomes differ in their capacity to hydrolyze these different classes of ubiquitinated cell proteins. Ubiquitinated short-lived proteins seem to differ from other ubiquitin conjugates in containing more branched ubiquitin chains (40) and perhaps also differ in their ease of unfolding or mode of delivery to the proteasome. Alternatively, proteasome activation may not be responsible for these different effects on degradation of long-lived proteins. Instead, PKG (unlike PKA) may promote the degradation of long-lived proteins by increasing their ubiquitination. In addition, although these two signaling systems both enhance the degradation of short-lived cell proteins, they may stimulate ubiquitination and hydrolysis of distinct populations of such proteins.

It is noteworthy that after a rise in cGMP, the cell’s content of ubiquitinated proteins increased, despite the enhanced proteasomal destruction of ubiquitinated proteins and total proteolysis by the UPS. Increases in the levels of ubiquitin conjugates are often interpreted as evidence of proteasome inhibition. However, the rapid rise in ubiquitin conjugates with PKG activation implies that the increase in protein ubiquitination must have exceeded the increase in their degradation. A similar rise in ubiquitin conjugates also occurs in atrophying muscles and during starvation despite the activation of 26S proteasomes and the increased proteolysis by the UPS (32). Thus, in many conditions where there is a global increase in ubiquitination, proteasomes may also be activated to handle the increased substrate load.

### Potential Applications in Combatting Neurodegenerative Diseases.

A large variety of neurodegenerative diseases result from the accumulation of misfolded, aggregation-prone proteins, which are generally found in intracellular inclusions conjugated with ubiquitin. This build-up of proteins likely represents a failure of the cell’s protein quality control machinery. In fact, there is growing evidence that such aggregates or microaggregates can impair the functioning of the 26S proteasomes (14, 20, 21), as was also shown here (Fig. 5) and previously (33) in a zebrafish model of tauopathy. The decrease in protein degradation should cause a further accumulation of misfolded proteins and eventually disrupt protein homeostasis. Therefore, drugs that enhance proteasome activity and promote the degradation of such potentially toxic proteins are an attractive approach to combat the progression of proteotoxic diseases. Accordingly, agents that raise cAMP promote the clearance of disease-associated Tau mutants in cells (13), and in a mouse model of frontotemporal dementia, where they not only increased proteasome activity but also lowered the levels of phospho-tau in the brain (14, 16). Similar effects of rolipram on proteasome activity and phospho-tau accumulation were observed here after treatment of the zebrafish model of tauopathy caused by overexpression of A152T-tau.

Surprisingly, raising cGMP with sildenafil had very similar effects in these zebrafish larvae as rolipram, not only in the tauopathy models but also in a Huntington’s disease model caused by the expression of a huntingtin exon 1 with a highly expanded polyQ sequence. Although PKA and PKG have very different physiological actions, their key shared property in these responses must be their capacity to enhance the degradation of short-lived misfolded proteins by the UPS. Remarkably, both sildenafil and rolipram, while enhancing proteasome activity and tau degradation, reduced neuronal death and the gross morphological abnormalities caused by overexpression of mutant tau in neurons. Furthermore, the treatments did not have any evident deleterious consequences, probably because they simply augment a physiological mechanism for protein quality control. While activation of PKG and PKA reduced the levels of the aggregated proteins, these structures are generally much larger than proteasomes. So, it remains unclear whether proteasomal digestion involves faster degradation of these misfolded proteins before they aggregate, or whether the proteasomes can extract ubiquitinated proteins from such aggregates for degradation. It also remains unclear whether these beneficial effects result solely through proteasome activation or through enhanced ubiquitination of the toxic proteins.

Thus, agents that raise cGMP in the central nervous system represent a promising new strategy to combat Alzheimer’s, Huntington’s, and other neurodegenerative diseases. In humans, the PDE5 inhibitors and the soluble guanylyl cyclase stimulators used here poorly cross the blood brain barrier. However, brain-penetrant PDE5 inhibitors (41) and soluble guanylyl cyclase stimulators (17) have been recently reported and inhibitors of other phosphodiesterases may also raise cGMP levels in parts of the CNS. Furthermore, patients with Alzheimer’s disease have lower levels of cGMP in their cerebrospinal fluid (42, 43) and greater expression of PDE5 in the temporal cortex (43) than healthy, age-matched controls. Thus, a reduction in cGMP signaling in the brain may contribute to the progression of Alzheimer’s disease in humans by decreasing ubiquitination, proteasome activity, and degradation of misfoldedb proteins.

The present findings and other’s prior observations on a mouse model of cardiomyopathy caused by an αB crystallin mutation (10, 44) demonstrate the therapeutic potential of treatments that raise cGMP to combat diverse proteotoxic diseases. Protein damage has also been reported to accompany other diseases, including cardiac failure and ischemia (45). The ability of PKG to enhance global protein degradation by the UPS may also contribute to its reported benefits in such conditions (46). Thus, the activation of PKG is a promising approach to combat the progression of a variety of presently untreatable proteotoxic diseases.

## Materials and Methods

For details on general methods, reagents, antibodies, proteasome purifications, proteasome activity assays, measurements of intracellular protein degradation, cell-free assays, statistics, and zebrafish husbandry and experiments, please refer to *SI Appendix*.

### Maintenance of Zebrafish Stocks and Transgenic Lines.

All zebrafish procedures were performed in accordance with the UK Animals (Scientific Procedures) Act with appropriate Home Office Project and Personal animal licenses and with local Ethics Committee approval. Studies were performed in accordance with ARRIVE guidelines.

### Data Availability.

All data in this report are available to all readers. The reagents used in this study, as well as further information, are available upon request from A.L.G.

## Supplementary Material

Supplementary File
