# Supplementary material for: cGMP via PKG activates 26S proteasomes and enhances degradation of proteins, including ones that cause neurodegenerative diseases
Source: Proc Natl Acad Sci U S A. 2020 Jun 8;117(25):14220–30. doi: 10.1073/pnas.2003277117 (PMC7321992; doi:10.1073/pnas.2003277117)
Supplement: Supplementary File [file pnas.2003277117.sapp.pdf]

PNAS
www.pnas.org

Supplementary Information for

cGMP via PKG activates 26S proteasomes and enhances degradation of proteins, including ones that cause neurodegenerative diseases

Jordan JS VerPlank[1]; Sylwia D Tyrkalska[2,3,4]; Angeleen Fleming[2,3,4]; David C Rubinsztein [2, 3]; Alfred L Goldberg, [1]
1.) Department of Cell Biology, Blavatnik Institute, Harvard Medical School, Boston, MA
2.) Department of Medical Genetics, Cambridge Institute for Medical Research, University of Cambridge, The Keith Peters Building, Cambridge Biomedical Campus, Hills Road, Cambridge CB2 0XY, UK.
3.) UK Dementia Research Institute, Cambridge Institute for Medical Research, University of Cambridge, The
Keith Peters Building, Cambridge Biomedical Campus, Hills Road, Cambridge CB2 0XY, UK
4.) Department of Physiology, Development and Neuroscience, University of Cambridge, Downing Street, Cambridge CB2 3DY, UK.

Corresponding Author:
Alfred Goldberg
alfred_goldberg@hms.harvard.edu

This PDF file includes:

Supplementary text (Materials and Methods)
Figures S1 to S10
SI References

www.pnas.org/cgi/doi/10.1073/pnas.2003277117

Supplementary Text

Materials and Methods

Reagents: Tadalafil, Sildenafil, Cinaciguat, BAY41-2272, KT-5823, and Rp-8-pCPT-cGMPs were purchased from Cayman Chemical, puromycin, cycloheximide, 1,10-phenanthroline and carbamoylcholine chloride from Sigma, Concanamycin A from Santa Cruz, Carfilzomib and Bortezomib from UBPBio, λ-phosphatase from New England Biosciences, L-[3,4,5-3H]-phenylalanine from American Radiolabeled Chemicals, PR-619 from Selleckchem, cyclic GMP from Promega, and PKG1α and Ubiquitin-amc from ThermoFisher Scientific. Rolipram and sildenafil used in zebrafish experiments were purchased from Tocris.

Cell Lines: All cell lines were obtained from ATCC. HEK293 and BJ5A cells, and C2C12 myoblasts, were cultured in DMEM supplemented with 10% FBS. C2C12 were differentiated into myotubes for 5 days in DMEM supplemented with 2.5% horse serum. SH-SY5Y cells were cultured in DMEM/F12 supplemented with 10% FBS.

Cell lysis and immunoblot: After treatments, cells were washed twice in cold PBS and then flash frozen on dry ice. The cells were lysed in the following buffer: 25 mM HEPES-KOH pH 7.5, 150 mM NaCl, 2.5 mM EDTA, 0.3% CHAPS, 10 mM N-Ethylmaleimide, 0.5 mM PMSF, 1 µM MG-132, 1 mM NaF, and 5 mM β-glycerophosphate. Lysates were centrifuged at 14,000 x g for 10 minutes and protein amount was determined with Detergent Compatible Bradford Reagent (ThermoFisher Scientific). The volumes of lysates were adjusted with lysis buffer

to ensure that all samples were of equal concentration, LDS Bolt sample buffer (Invitrogen) was added to a final concentration of 1X, and then samples were boiled for 5 minutes @ 95°C. SDS-PAGE was performed with 4-12% Bis-Tris Plus polyacrylamide gels (ThermoFisher Scientific). Proteins were transferred to either nitrocellulose (Protran; VWR) or PVDF (Immobilin FL; EMD Millipore) membranes and immunoblotting was performed using the following antibodies: Ubiquitin (VU1, Life Sensors), K48-linked polyubiquitin (D9D5, Cell Signaling), K63-linked polyubiquitin (D7A11, Cell Signaling), K11-linked polyubiquitin (2A3/2E6, EMD Millipore), p-PKA substrates (100G7E, Cell Signaling), pVASP (3114, Cell Signaling), PSMB5 (A303-847, Bethyl), PSMB1 (D-9, Santa Cruz), Rpn6 (D1T1R,Cell Signaling), PSMD2 (A303-854,Bethyl), Protein Kinase G (C8A4,Cell Signaling), LaminB1 (H-90, Santa Cruz), PKAα catalytic subunit (A-2,Santa Cruz), PSMA6 (A303-809, Bethyl), Puromycin (12D10, EMD Millipore), p53 (DO-1,Santa Cruz), PSMD1 (C-7, Santa Cruz), α1234567 (MCP231, Enzo), GAPDH (G9545, Sigma), β-Tubulin (AA2, EMD Millipore), pRpt3-Thr25 (a gift from J. Dixon (1), pRpt6-S120 (2), pRpn6-S14 generated as described previously (3). Visualization was performed with the Odyssey CLx infrared imaging system (LiCor) and quantified with ImageStudio.

Cell Transfection and plasmids: HEK293 cells were transfected with 0.5 μg/mL plasmid DNA using the PEImax reagent at a 3:1 ratio of PEImax to DNA. Media was replaced 5 hours after transfection. The plasmids used in this study were acquired from Addgene: PKAα catalytic subunits (45521) and PKG1α (16392).

siRNA-mediated knockdown of PRKG1: ON-TARGET plus SMARTPOOL siRNA for PKG and Scramble were purchased from Dharmacon. Transfection was performed in SH-SY5Y cells for 72 hours with the Dharmafect 1 transfection reagent per manufacturer's instructions.

Subcellular fractionation: Cytoplasmic and nuclear fractions were produced as previously described (4), with minor changes. Briefly, cells were suspended in STM buffer (50 mM Tris-HCl pH 7.5, 250 mM sucrose, 5 mM $MgCl_2$, 1 mM ATP, 0.5 mM PMSF, 25 nM Calyculin A, 1 mM NaF, and 1 mM β-glycerophosphate), incubated on ice for 30 minutes and then passed 25 times through a 21-gauge needle. The crude lysate was centrifuged at 800 x g for 15 minutes at 4^oC. The supernatant (cytosol plus mitochondria) was transferred to a new tube and the pellet (nucleus plus cellular debris) was resuspended in STM buffer, vortexed and centrifuged at 500 x g for 15 minutes at 4^oC. The supernatant (cell debris) was disposed and the pellet (nuclear fraction) was washed once more in STM buffer (1,000 x g for 10 minutes) and then resuspended in NET buffer (25 mM HEPES-KOH pH 7.5, 5 mM $MgCl_2$, 100 mM NaCl, 10% glycerol, 1 mM ATP, 0.5 mM PMSF, 25 nM Calyculin A, 1 mM NaF, and 1 mM β-glycerophosphate). The crude nuclear fraction was incubated for 30 minutes on ice, sonicated, and then centrifuged at 9,000 x g for 30 minutes at 4^oC. The supernatant was collected as the nuclear fraction. The cytosolic/mitochondrial fraction was centrifuged at 10,000 x g for 10 minutes to remove the mitochondria and sonicated identically as the nuclear fraction. 5M NaCl was added to the cytosolic fraction to a final concentration of 100 mM, to make it equimolar with the nuclear fractions.

Relative protein concentration of cytosolic and nuclear fractions was measured by Bradford assay (ThermoFisher Scientific) and buffer was added to each sample to bring the fractions to the same relative protein concentration. 26S proteasomes were then purified from each fraction using the UBL-method (5). Because lower amounts of 26S proteasomes were purified from the nuclear fractions of SH-SY5Y cells, 26S proteasomes from the cytosolic fractions were diluted to the concentration obtained from the nuclear fractions. An equal amount of proteasomes from each fraction was used in all assays.

Activation of PKG and ubiquitination in cytosolic fractions: The cytosolic fraction from HEK293 cells was prepared in STM buffer as described above, plus IBMX (45 μM). The reaction was prepared on ice in STM buffer plus 0.5 mM EGTA and the following agents were added as indicated: cGMP (Promega), Bortezomib (UBPbio)(1μM), PR-619 (SelleckChemical)(10μM), 1,10-phenanthroline (Sigma)(250μM). After the addition of all agents, the samples were incubated at RT for 5 minutes, and then incubated at 37°C for 30 minutes. The reaction was stopped by the addition of Laemmli buffer and boiling.

Measuring deubiquitination of proteins in cytosolic fractions: HEK293 cells were treated for 30 minutes with 1 μM Bortezomib, and then cytosolic fraction was prepared in STM buffer plus IBMX. The reaction was prepared on ice in STM buffer plus 0.5 mM EGTA plus Bortezomib (UBPbio)(1μM), Tak243 (1μM), and cGMP (1μM) where indicated. The samples were vortexed, incubated at RT for 5 minutes, and then incubated at 37°C for the indicated times. The reaction was stopped by the addition of Laemmli buffer and boiling.

Measuring activity of deubiquitinases in cell lysates: SH-SY5Y cells were treated for 30 minutes with 100 nM of tadalafil, cinaciguat, or both and then lysed as described for measurements of proteasome activity. Active site modification of DUBs by HA-Ub-VS and hydrolysis of Ub-amc were performed as previously described (6).

Degradation of long- or short-lived cell proteins: The degradation rate of long- or short-lived cell proteins was measured after labeling with [^{3}H]phenylalanine for different lengths of time, and the conversion of radiolabeled cell proteins to TCA-soluble radiolabeled amino acids in the media was assayed as described (7).

Affinity purification and activity measurements of 26S proteasomes: 26S proteasomes were purified from cells in culture and zebrafish larvae using the ubiquitin-like domain (Ubl) method described previously (5). Cells were lysed in 25 mM HEPES-KOH pH 7.5, 100 mM NaCl, 5 mM $MgCl_2$, 1 mM ATP, 1 mM DTT, and 0.5 mM PMSF plus the phosphatase inhibitors, 25nM Calyculin A, 10mM NaF, and 20mM β-glycerophosphate. Proteasomal peptidase activities were measured as described previously (8), and ATPase activity was assayed using the EnzCheck Phosphate Assay Kit (ThermoFisher Scientific). The ATPase assay was performed at 32 ^{o}C with 2 nM 26S proteasomes in 100 μL of the reaction buffer: 50 mM Tris (pH 7.5), 5 mM $MgCl_2$, 1 mM ATP, 1 mM DTT, 2% glycerol, and 0.1 mg/mL BSA. The rate of ATP hydrolysis was measured from the linear portion of the reaction. The fluorescent protein EOS was used to assay degradation of ubiquitinated proteins by 26S proteasomes. EOS was expressed,

purified, ubiquitinated, and fractionated to achieve substrates of some uniform style as described previously (9). Polyubiquitinated EOS conjugated to ubiquitin chains containing 5-10 ubiquitins (50 nM) was incubated with 26S proteasomes (5 nM) in 20 μL reaction buffer (50 mM Tris pH 7.5, 5 mM $MgCl_2$, 1 mM ATP, 1 mM DTT, 10 nM Calyculin A, 1 mM NaF, 1 mM β-glycerophosphate, and 0.1 mg/mL BSA in 384-well plates. Degradation of EOS was performed at 32 ^oC and was followed by the loss of EOS fluorescence (excitation 490 nm; emission 525 nm).

Detecting assembled 26S proteasomes by Native PAGE: Cells were lysed by sonication in APB (25 mM Hepes-KOH pH 7.5, 150 mM NaCl, 5 mM $MgCl_2$, 10% glycerol, 1 mM ATP) and run on 3-8% gradient NuPAGE Tris Acetate Gels (Life Technologies) in Tris-Glycine Native buffer (Thermo) supplemented with 5 mM $MgCl_2$ and 1 mM ATP for 4 hours at 135 V at 4°C. For western blot analysis, gels were soaked for 15 minutes at room temperature in transfer buffer containing 48 mM Tris, 39 mM glycine and 0.04% SDS and then transferred to PVDF membranes in transfer buffer overnight at 25V at 4°C.

Dephosphorylation of 26S proteasomes with lambda phosphatase: 26S proteasomes were purified by the UBL-method and incubated with either phosphatase inhibitors (Calyculin A, 25 nM; NaF, 1 mM; β-glycerophosphate, 1 mM) or lambda phosphatase for 60 minutes at 30 ^{O}C as previously described previously (10). Peptidase activity was then assayed as described above.

Phosphorylation of 26S proteasomes by PKG: Proteasomes affinity-purified by the UBL-method from BJ5A cells were incubated with the indicated concentrations of PKG1α (ThermoFisher Scientific) for 60 minutes at 30 ^{o}C in the buffer: 50 mM Tris pH 7.5, 5 mM $MgCl_2$, 1 mM ATP, 10 mM cGMP, 10 nM Calyculin A, 1 mM NaF, and 1 mM β-glycerophosphate. Peptidase activity was then measured as described above in the presence of the PKG inhibitor KT5823 (500 nM).

Degradation of puromycyl polypeptides: SH-SY5Y were exposed to puromycin (5 µg/mL) for 1 hour. The cells were then washed twice with media containing cycloheximide (100 µg/mL) and new media was then added containing cycloheximide and tadalafil (100 nM) or DMSO. At the indicated time points, cells were collected and flash frozen on dry ice.

Maintenance of zebrafish stocks and transgenic lines: All zebrafish procedures were performed in accordance with the UK Animals (Scientific Procedures) Act with appropriate Home Office Project and Personal animal licences and with local Ethics Committee approval. Studies were performed in accordance with ARRIVE guidelines.

Zebrafish were bred and maintained under standard conditions at 28.5 ± 0.5 °C on a 14 h light: 10 h dark cycle. Embryos were collected in embryo medium (EM) (5 mM NaCl, 0.17 mM KCl, 0.33 mM $CaCl_2$, 0.33 mM Mg_2SO_4, 5 mM HEPES) and staged according to established criteria (11). Generation of the Rho::EGFP-TauWT (Rho::EGFP-tau^{cu7}), Rho::EGFP-HD71Q (Rho::EGFP-HTTQ71^{cu5}) and Rho::EGFP (Rho::EGFP^{cu3}) was previously described (12, 13). Rho::EGFP-

TauP301L (Rho::EGFP-tauP301L^{cu12}) not previously reported, was generated as described for Rho::EGFP-TauWT (12). The UAS::Dendra-TauA152T (Tg(UAS:Dendra2-Hsa.MAPT_A152T,myl7:EGFP^{cu10})) line was generated as previously reported (14). The pan-neuronal Gal4 driver line (hereafter referred to as PanN::Gal4VP16) was a kind gift from Herwig Baier (identified as line s1101tEt in the original publication) (15). The ubiquitous Gal4 driver line EIF1α::Gal4VP16 (Xia.Eef1a1:GAL4-VP16^{cu11}) was made in-house, as previously described (14).

Compound treatment in zebrafish transgenic lines and Dendra-tau phenotype determination: Embryos were collected in EM and reared at 28.5 °C. Larvae from the outcross of Rho::EGFP-TauWT, Rho::EGFP-TauP301L and Rho::EGFP-HD71Q with wild type fish were treated from 1 day post fertilization (d.p.f.) with EM containing 0.03% phenylthiourea (PTU) to prevent pigmentation. At 3 d.p.f, larvae were screened for EGFP expression in the rod photoreceptors and then removed from PTU and reared in EM. Larvae were treated either with 1 µM or 10 µM sildenafil, 3 µM or 30 µM rolipram or 0.1 % DMSO as a control from 4 to 9 d.p.f. Compounds and EM were refreshed daily. At 9 d.p.f., larvae were anaesthetized, then fixed in 4% PFA in PBS and processed for the analysis of rod photoreceptor numbers or mutant HD aggregate quantification.

Larvae expressing Dendra-TauA152T in the central nervous system (PanN::Gal4VP16 driver) were reared in EM until 1 d.p.f. then treated with 0.1 % DMSO, 1 µM or 10 µM sildenafil or 3 µM or 30 µM rolipram either from 1 to 3

d.p.f. Drugs and medium were replenished daily. The percentage of larvae with different morphological phenotypes was quantified at 3 d.p.f. Phenotypes were classified either as normal; mild (when slight torsion of dorsal spine was observed or when head axis was not aligned to the dorsal spine but the larvae could swim straight); or severe (when fish showed a complete torsion of whole body in 'U'-shape) as previously described (14).

Western blotting from zebrafish larvae: Tissue samples from Dendra-TauA152T positive larvae, with the expression of Dendra-TauA152T in central nervous system at 3 d.p.f., or Rho::EGFP-TauWT and Rho::EGFP-TauP301L larvae at 9.d.p.f. were lysed on ice with lysis buffer containing 1% octylglucoside, complete protease cocktail and PhosSTOP tablets (SIGMA). Tissue was homogenized by sonication and lysates were centrifuged at 7000 rpm for 1 min at 4 ^{0}C. The concentration of protein in the lysates was measured using the bicinchoninic acid (BCA) protein assay reagent (Pierce) with BSA as the standard. Supernatants were diluted in 2x Laemmli Buffer at a 1:1 dilution, resolved by sodium dodecyl sulphate polyacrylamide gel electrophoresis (12%) and transferred to PVDF membranes. The membranes were blocked with PBST containing 5% non-fat dry milk or 3% BSA and were then incubated overnight at 4 ^{0}C with primary antibodies diluted in 5% non-fat dry milk, 3% BSA or PBST. Membranes were washed three times in PBST for 10 min each time, incubated for 1 h at room temperature with 1:5000 dilution of horseradish peroxidase-conjugated secondary anti-mouse or anti-rabbit antibodies (DAKO) in PBST, and

washed three times for 10 min each. Immunoreactive bands were then detected using ECL (GE Healthcare Bioscience). Quantification of proteins was performed using ImageJ (FIJI) software. The following antibodies were used: mouse anti-tau (1:1000; Abcam), mouse anti-PHF1 (1:100; a kind gift from Dr Peter Davies, Albert Einstein College of Medicine of Yeshiva University, NY), mouse anti-ZPR3 – anti-rod-specific rhodopsin (1:500; ZIRC), mouse anti-ZPR1 anti-cone specific arrestin-3a (1:250; ZIRC) and mouse anti-Tubulin (1:1000; Sigma).

Imaging and quantification of photoreceptors or huntingtin aggregates in central retina of zebrafish: 10 μm transverse cryosections were cut across the zebrafish retina of Rho::EGFP-TauWT, Rho::EGFP-TauP301L or Rho::EGFP-HD71Q using a Bright cryostat. Sections were examined to identify the optic nerve head (central retina) and 5 sections at the level of the optic nerve head were imaged using a Zeiss Axioplan2 fluorescent microscope equipped with a QImaging Retiga 2000 R digital camera. Fluorescence images of the GFP-signal were then used to determine the number of photoreceptors or 71Q HD aggregates.

RNA preparation and quantitative reverse transcription polymerase chain reaction: Pools of 15 embryos of Rho::EGFP fish were collected at 9 d.p.f. Total RNA was isolated using RNeasy-Plus Mini-Kit (Qiagen) according to the manufacturer's instructions. 100 ng RNA was then used in One-Step qRT-PCR combining cDNA synthesis by specific-primed reverse transcription and real-time

PCR reaction according to the manufacturer protocols (Invitrogen) using TaqMan Enzyme mix and customized TaqMan gene-specific primers for GAPDH (as the housekeeping gene) and GFP (from Applied Biosystem). All samples were run in triplicate and were analyzed on a StepOne Plus Real Time PCR System and StepOne Software V.2.1 (Applied Biosystem, Life technologies). Relative gene expression was normalized to GAPDH controls and assessed using 2-ΔΔCT method.

Proteasome activity assay in lysates of zebrafish larvae: Dendra-TauA152T positive larvae were treated from 24 hours post fertilization (h.p.f.) with 1 μM or 10 μM sildenafil and 3 μM or 30 μM rolipram (1% DMSO as a control). After 24 h of drug treatment, larvae were culled and tissue homogenized in 50 mM Tris (pH 7.5), 1 mM DTT by brief sonication (twice for 10s). The assay was run with three independent samples per treatment, and each sample was analyzed in triplicate. Tissue lysates were centrifuged at 16900 g for 10 minutes at 4 ^{0}C. Samples normalized for protein concentration (40 mg total protein) were loaded into a 96-well plate. Chymotrypsin-like activity of the 26S proteasome was monitored using a FLUOstar Omega fluorometer (BMG Labtech) with Omega.LNK software. The reaction was started by the addition of 100 mM Suc-LLVY-AMC (Enzo) diluted in homogenizing buffer at the zero time point. The activity was assayed by measuring the fluorescence intensity during 4.5 h at 28 ^{0}C, in 5-min intervals (excitation 355 nm; emission 460 nm).

Quantification of Dendra-tau clearance: Crosses of UAS::Dendra-tauA152T responder fish with EIF1α::Gal4VP16 driver fish were performed to produce offspring with ubiquitous but mosaic expression of the transgene to allow visualization of individual neurons in the spinal cord. Embryos were sorted at 24 h.p.f. to identify fluorescent Dendra-TauA152T expressing individuals. Photoconversion of individual spinal cord neurons was performed at 2 d.p.f., as previously described (14). Briefly, larvae were anaesthetized by immersion in 0.2 mg/ml 3-amino benzoic acid ethyl ester (MS222) and mounted in 1% low melting agarose in embryo medium. Photoconversion of Dendra was performed by UV irradiation (405 nm) for 3 seconds using Bleachpoint tool and a confocal microscope Leica SP8 at x3 magnification with a 40x objective. Digital fluorescent images of individual neurons were taken immediately after Dendra photoconversion and at 6-h. The images were then analyzed using ImageJ by selecting regions of interest around each neuron and fluorescent intensity was measured using ´ROI´ and Integrated Density functions. To monitor Dendra-TauA152T clearance, the fluorescent intensity of each cell was quantified at each time point and expressed as a percentage of the initial fluorescent intensity, immediately after photoconversion.

Statistical analysis: Data are shown as mean ± SEM and were analyzed by analysis of variance (One-way ANOVA) and Tukey multiple range test, Dunnett or Bonferroni post-hoc tests to determine differences between multiple groups in western blot densitometry analysis, intracellular protein degradation

measurements, proteasome activity assays, photoreceptor counts, quantification of number of huntingtin aggregates and gene expression analysis. The differences between two sample groups were analyzed by the Student´s *t*-test in comparing of time points in different treatments in Dendra photoconversion experiments as well as activity assays with purified proteasomes. The differences between the phenotypes in Dendra-TauA152T larvae were analyzed by Chi-square test. Two-way analysis of variance (Two-way ANOVA) was used to determine the differences between the plots of two different categorical independent variables in proteasome activity assays. Asterisks represent *p*-values, *$p \leq 0.05$, ** $p \leq 0.01$, *** $p \leq 0.001$.

Supplemental Figure 1

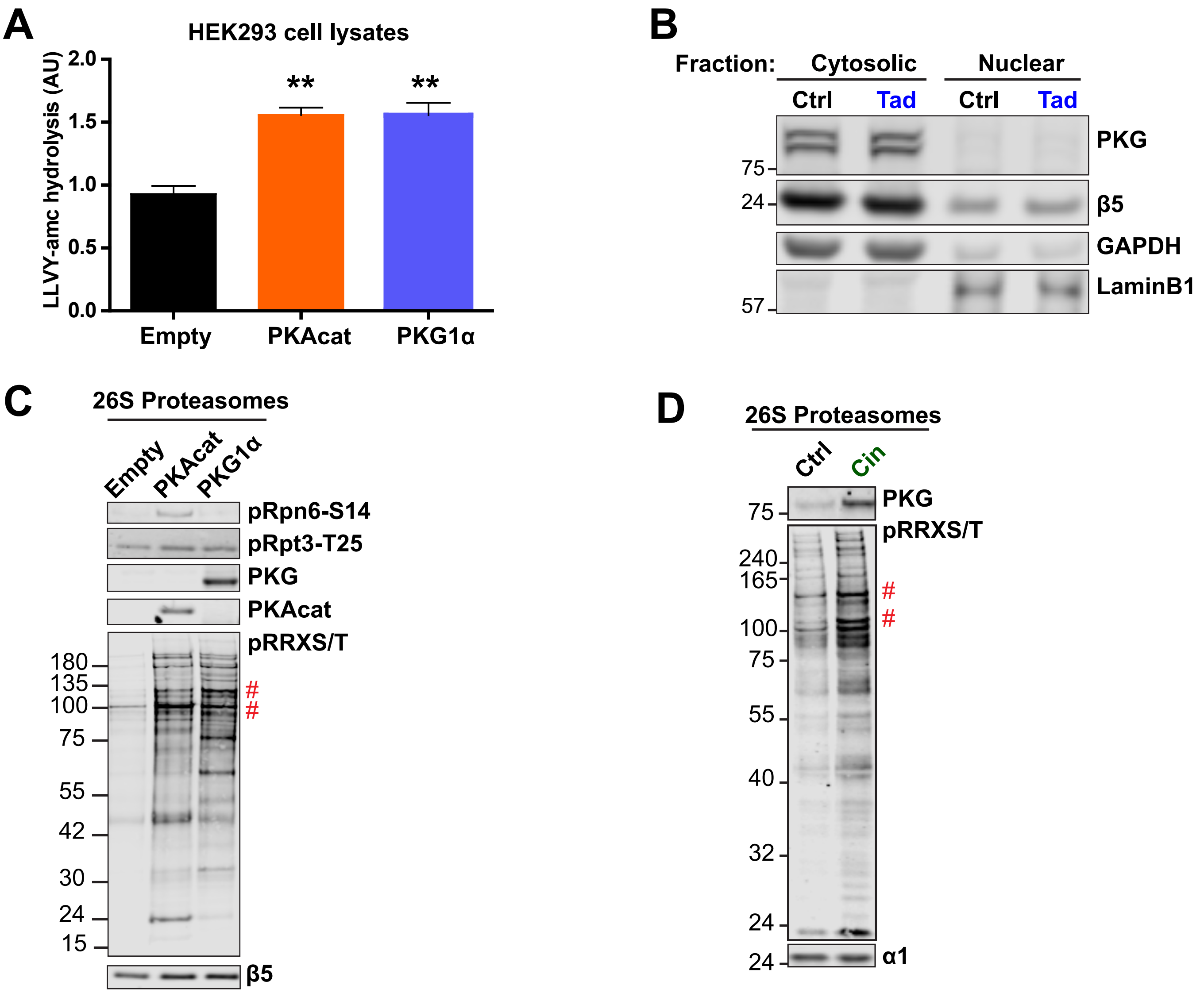

Supplemental Figure 1: PKG is predominantly localized to the cytosol in SH-SY5Y cells and co-purifies with 26S proteasomes in HEK293 cells with overexpression of PKG or treatment to raise levels of cGMP.

A.) Proteasomal chymotrypsin-like activity is increased by transient overexpression of PKG or the catalytic subunit of PKA. 24 hours after transfection with the indicated vectors, proteasomal chymotrypsin-like activity was measured in the cell lysates with suc-LLVY-AMC. n=3. Averages +/- SEM are shown. One-way ANOVA with Dunnett multiple comparison test. **p≤.0.01.

B.) PKG is predominantly localized to the cytosol. SH-SY5Y cells were treated with DMSO or tadalafil for 30 minutes and then separated by differential centrifugation into cytosolic and nuclear fractions. Equal amounts of protein from each fraction were analyzed by SDS-PAGE and western blot for PKG, the 20S proteasome subunit β5, GAPDH (cytosol), and LaminB1 (nucleus). Representative western blots from one of two experiments are shown.

C.) 26S proteasomes purified from HEK293 cells overexpressing PKG exhibit increased levels of co-purifying PKG and phosphorylated proteins. However, PKG overexpression did not increase the levels of phosphorylated Rpn6-S14, the proteasome subunit phosphorylated by PKA, or Rpt3-Thr25, the proteasome subunit phosphorylated by DYRK2. HEK293 cells stably over-expressing the proteasome subunit Rpn11 with both his-and biotin-tags (Rpn11-HTBH) were transfected with PKG, PKA catalytic subunit, or an empty vector. 24 hours after transfection, 26S proteasomes were purified via the biotin tag, and analyzed by SDS-PAGE and western blot with the indicated antibodies. Representative western blots of one of two proteasome purifications are shown.

D.) Increased levels of PKG co-purify with 26S proteasomes from HEK293 cells treated with cinaciguat, an activator of soluble guanylyl cyclase. HEK293 (Rpn11-HTBH) cells were exposed to DMSO or cinaciguat for 1 hour and then proteasomes were purified as in Supplemental Figure 1C. Representative western blots are shown for one of two proteasome purifications.

Supplemental Figure 2

A

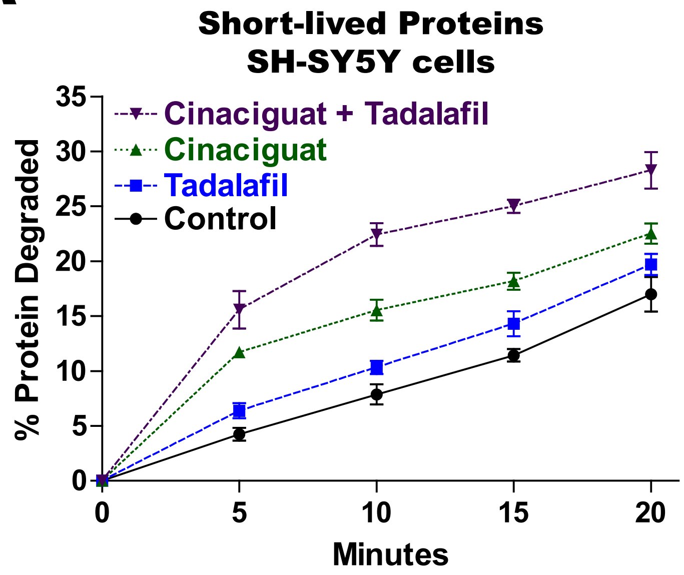

Supplemental Figure 2: Agents that raise cGMP stimulate the breakdown of short-lived proteins

A.) The breakdown of short-lived proteins was increased by exposure of SH-SY5Y cells to tadalafil (100 nM) or cinaciguat (100 nM). Combining the two agents to even further raise cGMP resulted in a greater increase in the degradation of these short-lived proteins. SH-SY5Y cells were pulsed for 20 minutes with 3H-Phenylalanine, and then protein degradation was measured in the presence of cycloheximide by following the conversion of radiolabeled proteins to peptides in the media. The indicated pharmacological agents were added to the media after the radiolabeling of cell proteins. n=4. Averages +/- SEM are shown.

Supplemental Figure 3

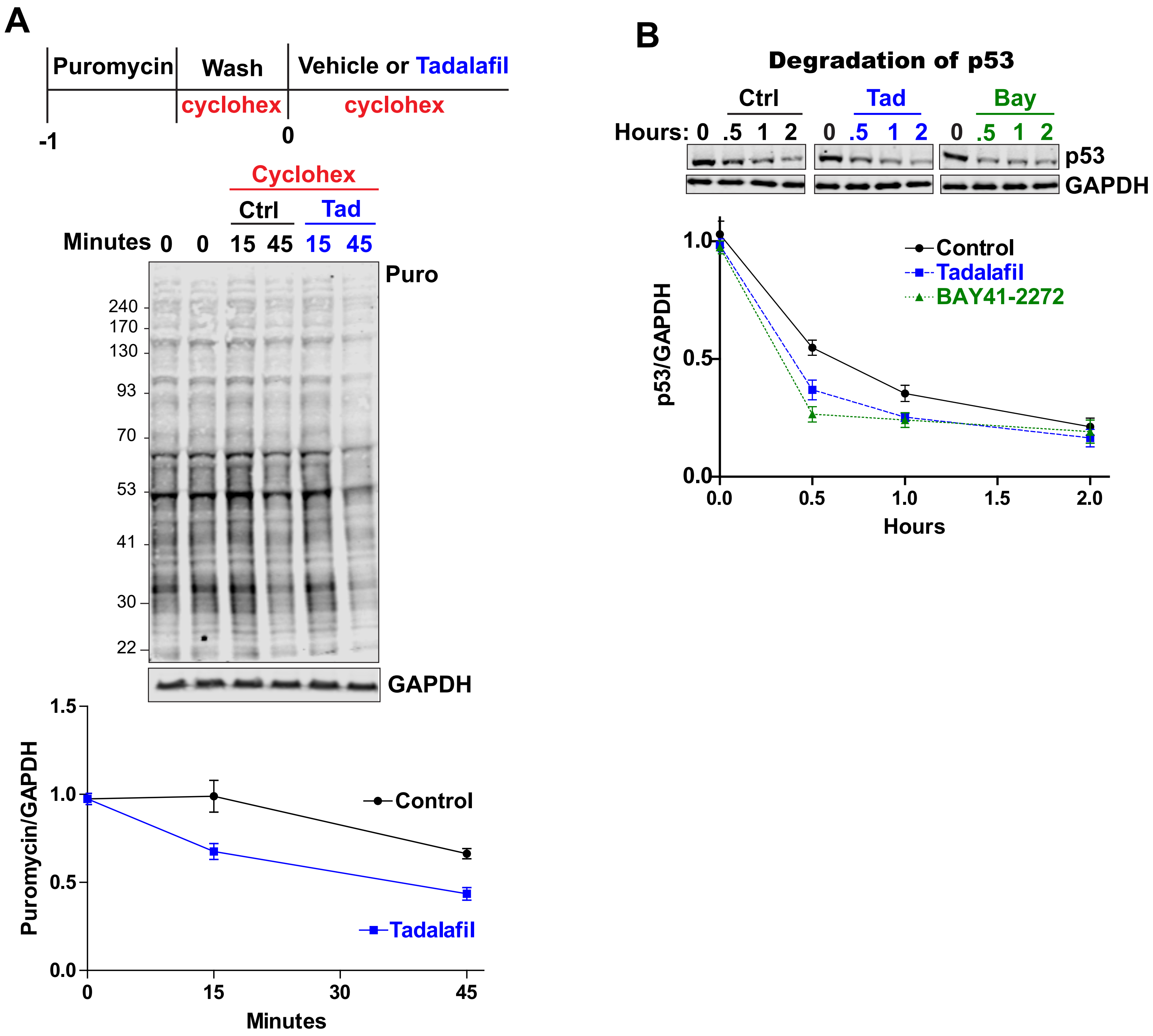

Supplemental Figure 3: Pharmacological agents that raise cGMP stimulate the degradation of proteins damaged by incorporation of puromycin and p53.

A.) Puromycin-containing polypeptides were degraded more rapidly in cells treated with tadalafil. To generate incomplete proteins, SH-SY5Y cells were exposed for 1 hour to puromycin (5 µg/mL), which is incorporated into newly synthesized proteins and causes premature termination of their translation. The degradation of these puromycin-containing polypeptides was then followed in the presence of cycloheximide (150 µg/mL) with or without the addition of tadalafil. The puromycin-containing polypeptides were detected with an antibody against puromycin and the signal throughout the entire lane was quantified. Representative western blots of one of three experiments are shown. n=3. Averages +/- SEM.

B.) The degradation of p53 was faster when cGMP is raised with tadalafil or BAY41-2272. SH-SY5Y cells were exposed to cycloheximide to block protein synthesis and pharmacological agents to raise cGMP. The amounts of p53 in the cell lysates were analyzed by western blot. Representative western blots are shown from one of three experiments. Averages +/- SEM.

Supplemental Figure 4

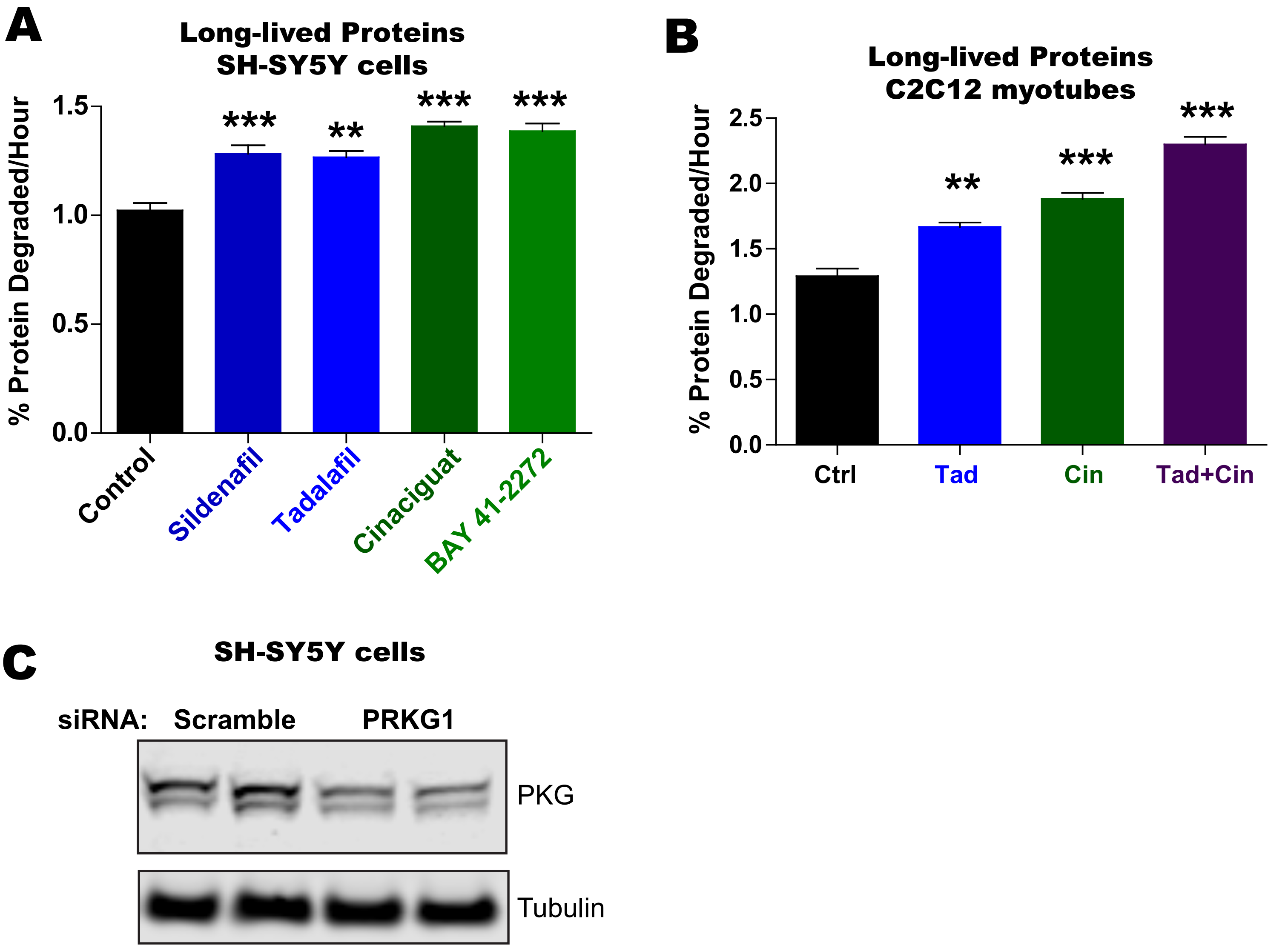

Supplemental Figure 4: Agents that raise cGMP enhance the degradation of long-lived proteins

A.) The degradation of long-lived proteins is increased in SH-SY5Y cells by exposure to the indicated PDE5 inhibitors or soluble guanylyl cyclase stimulators. Cells were exposed to 3H-Phenylalanine for 16 hours, chased with excess non-labeled phenylalanine for 2 hours, and then the conversion from radiolabeled proteins to labeled-amino acids in the media was measured. These agents were added to the media after the chase period. n=3. Averages +/- SEM are shown. One-way ANOVA with Dunnett multiple comparison test. $^{**}p \leq 0.01$. $^{***}p \leq 0.001$.

B.) Tadalafil and cinaciguat increased the degradation of long-lived proteins in mouse myotubes. Combining the two agents resulted in an even larger increase in the degradation of these proteins. C2C12 cells were differentiated into myotubes for 5 days prior to labeling with 3H-Phenylalanine. n=3. Averages +/- SEM are shown. One-way ANOVA with Dunnett multiple comparison test. $^{**}p \leq 0.01$. $^{***}p \leq 0.001$.

C.) PKG protein levels are reduced by approximately 50% with siRNA-mediated knockdown of PRKG1 for 48 hours. Representative western blots from one of three experiments are shown.

Supplemental Figure 5

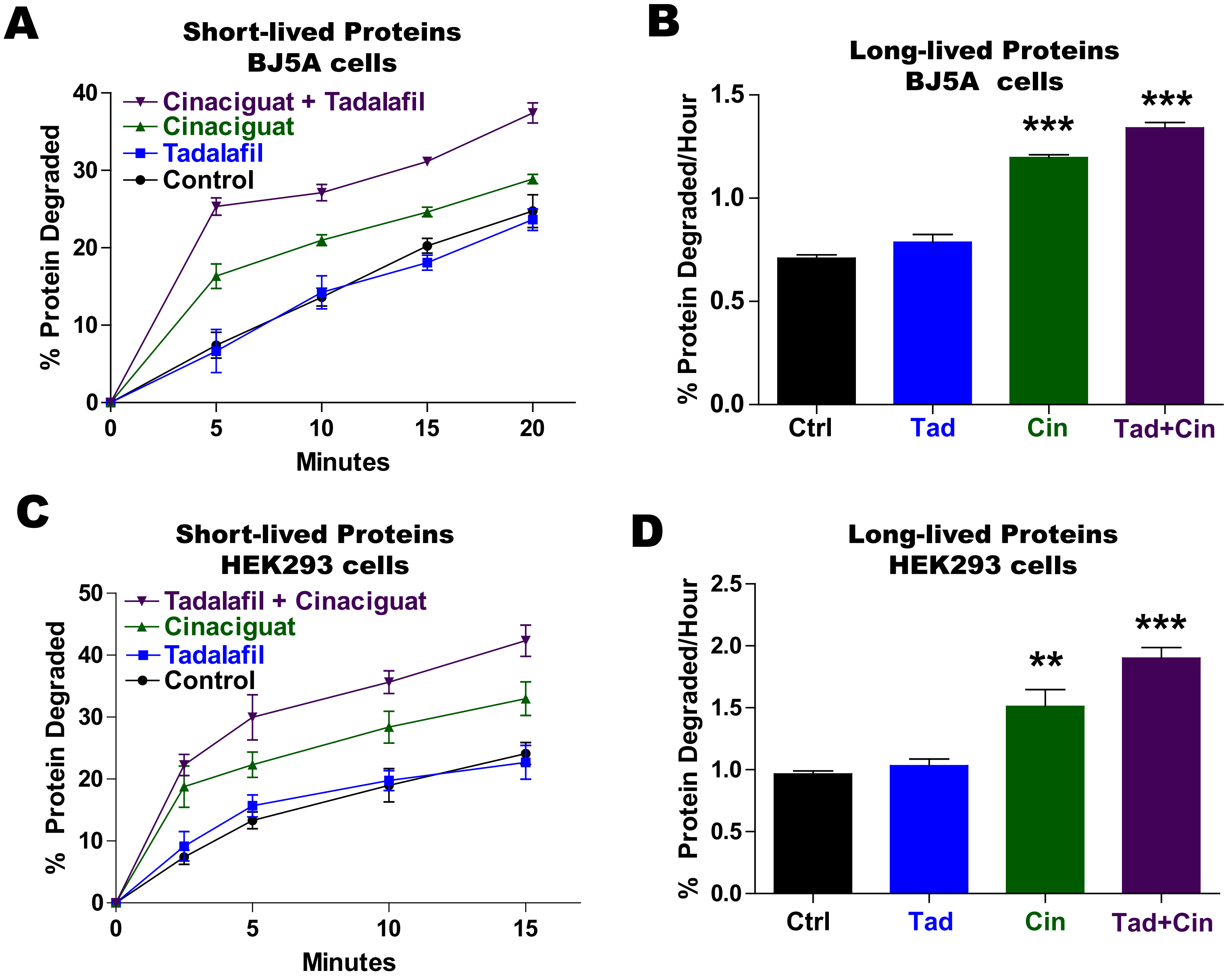

Supplemental Figure 5: In BJ5A human fibroblasts and HEK293 cells, raising cGMP with cinaciguat stimulates total protein breakdown and combining cinaciguat with tadalafil causes an even greater increase in protein breakdown.

A.) The breakdown of short-lived proteins is increased by exposure of BJ5A cells to cinaciguat (100nM), but not tadalafil (100 nM). This lack of stimulation by tadalafil is likely due to a low rate of cGMP synthesis. Combining cinaciguat and tadalafil to raise cGMP even further resulted in a greater increase in the degradation of the short-lived proteins. The degradation of short-lived proteins was performed as in Figure 3. n=4. Averages +/- SEM are shown.

B.) Cinaciguat increase the degradation of long-lived proteins in BJ5A cells and combining it with tadalafil resulted in an even larger increase in the degradation of this class of proteins. Experiment performed as in Figure 3. n=3. Averages +/- SEM are shown. One-way ANOVA with Dunnett multiple comparison test. ***$p \leq .001$.

C.) The breakdown of short-lived proteins is increased by exposure of HEK293 cells to cinaciguat (100nM), but not tadalafil (100 nM). Combining cinaciguat and tadalafil to raise even further cGMP resulted in a greater increase in the degradation of the short-lived proteins. The degradation of short-lived proteins was assayed as in Figure 3 and Supplemental Figure 2. n=4. Averages +/- SEM are shown.

D.) Cinaciguat increases the degradation of long-lived proteins in HEK293 cells and combining it with tadalafil caused an even larger increase in the degradation of these proteins. Experiment was performed as in Figure 3. n=3. Averages +/- SEM. One-way ANOVA with Dunnett multiple comparison test. **$p \leq 0.01$. ***$p \leq .001$.

Supplemental Figure 6

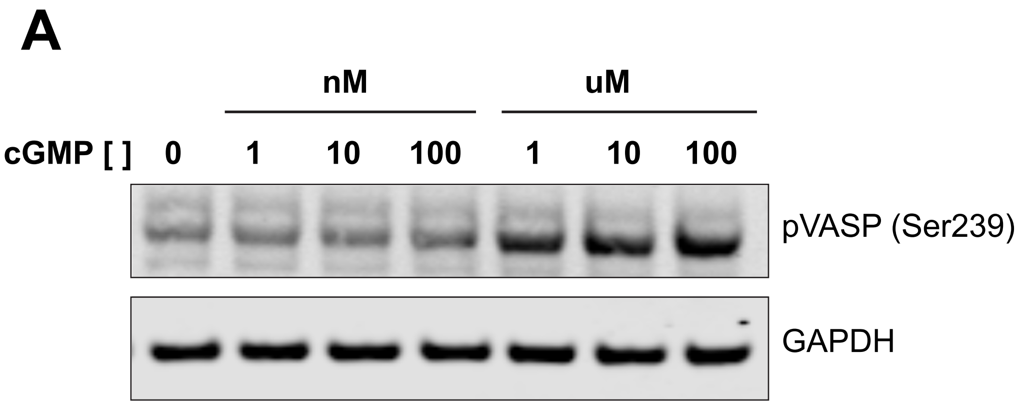

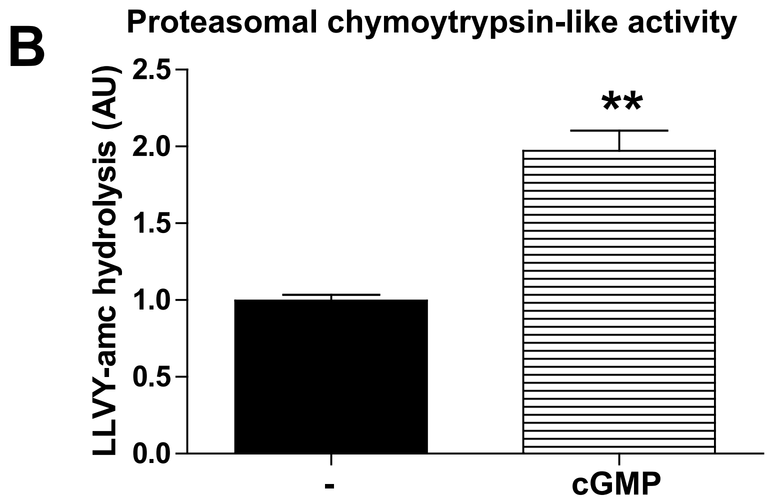

Supplemental Figure 6: Adding cGMP to cytosolic extracts from HEK293 cells activates PKG and proteasomal peptidase activity.

A.) Adding cGMP to cytosolic extracts of HEK293 cells increases the phosphorylation of VASP (Ser239), a well-characterized PKG substrate. HEK293 cells were lysed in a hypotonic buffer, and cytosolic extract was prepared by pelleting the nucleus (15 minutes at 800 x g) and then the mitochondria (10 minutes at 10,000 x g). After the addition of cGMP at the indicated concentrations, the extracts were incubated for 30 minutes at 37oC. The reactions were stopped by the addition of Laemmli buffer and boiling. Representative western blots form one of two experiments are shown.

B.) Incubating cytosolic extracts with 1 μM cGMP for 30 minutes at 37oC increases proteasomal chymotrypsin-like activity. n=4. Averages +/- SEM. Student's T test. **p≤ 0.01.

Supplemental Figure 7

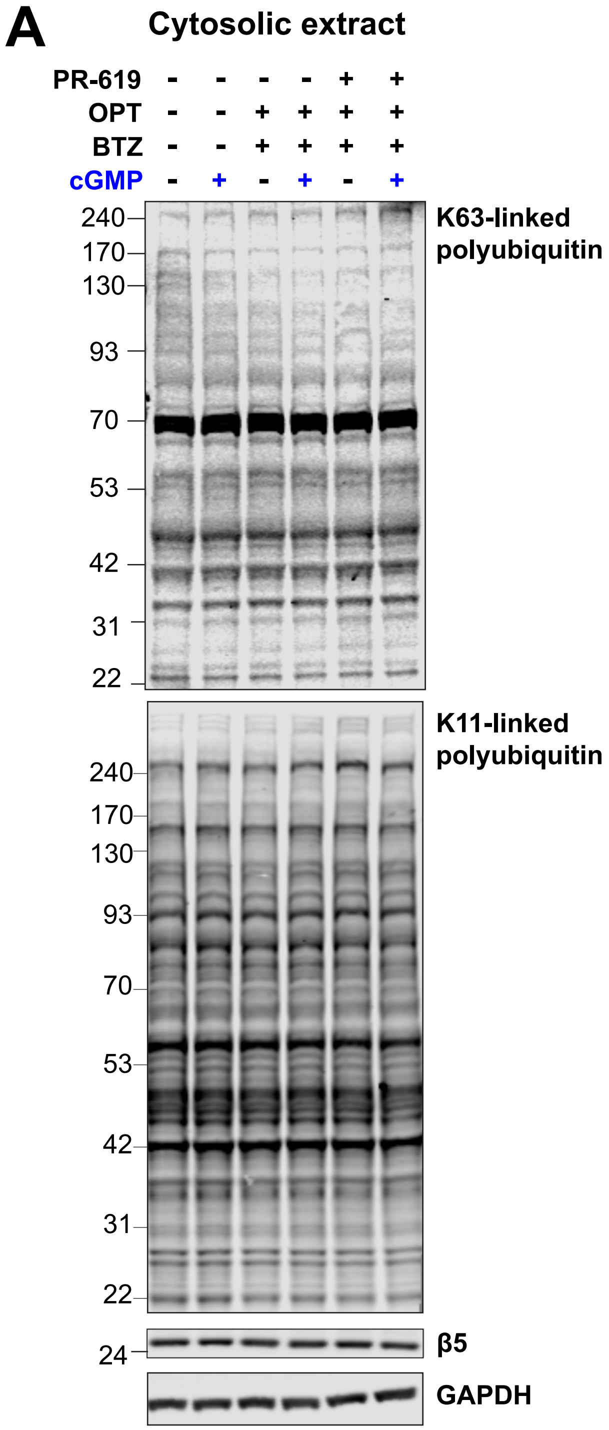

Supplemental Figure 7: Adding cGMP to cytosolic extracts from HEK293 cells does not change the levels of K63- or K11-linked polyubiquitinated proteins

A.) Incubating cytosolic extracts with 1 μM cGMP as in Figure 4D does not increase the levels of K63- or K11-linked polyubiquitinated proteins. Representative western blots of one of four experiments is shown.

Supplemental Figure 8

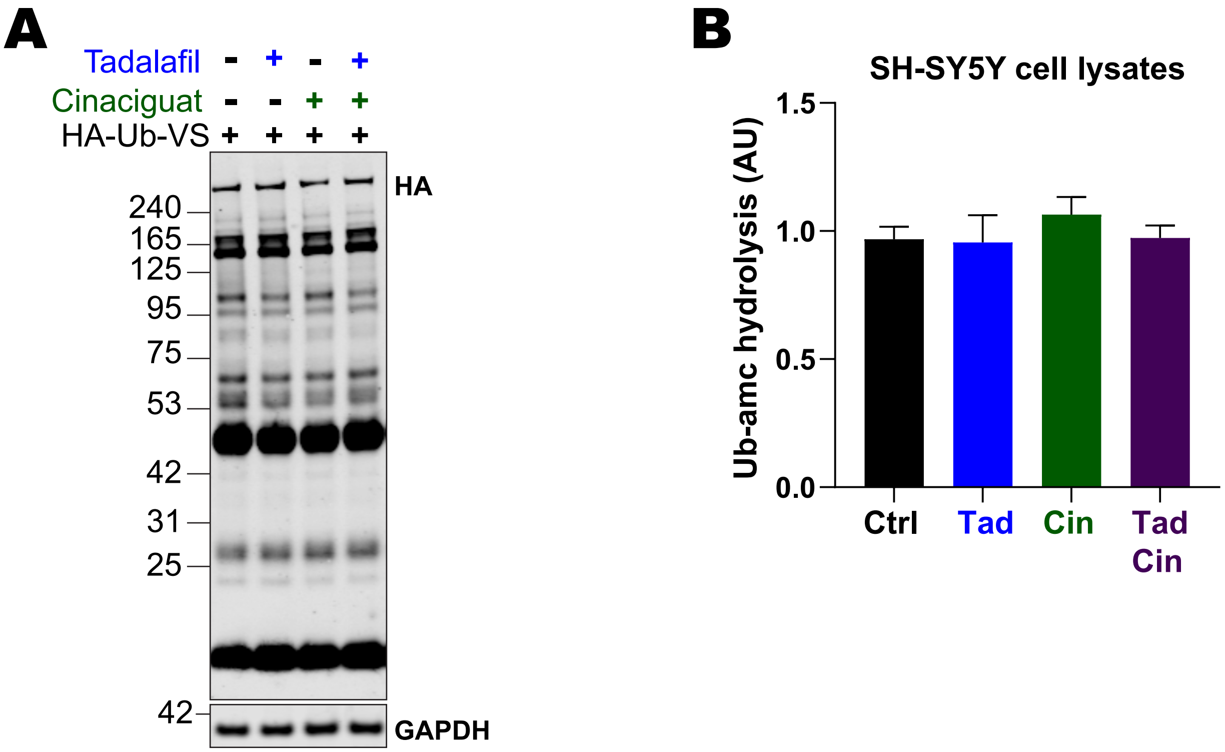

Supplemental Figure 8: Raising cGMP in SH-SY5Y cells does not change the activity of deubiquitinases

A.) Treating SH-SY5Y cells with tadalafil, cinaciguat, or both, does not change the amount of active deubiquitinases modified by HA-Ub-VS. The cells were treated for 30 minutes with the indicated agents, and then the lysates were incubated at RT for 30 minutes with 250 nM HA-Ub-VS. The reactions were stopped by the addiation of Laemmli buffer and boiling. A representative western blot from one of three experiments is shown.

B.) Raising cGMP in SH-SY5Y cells does not change the amount of deubiquitinase activity in the lysates. After 30 minutes of treatment with the indicated agents, the hydrolysis of Ubiquitin-AMC was measured at 37oC during the linear phase of the reaction. n=4. Error bars indicate SEM.

Supplemental Figure 9

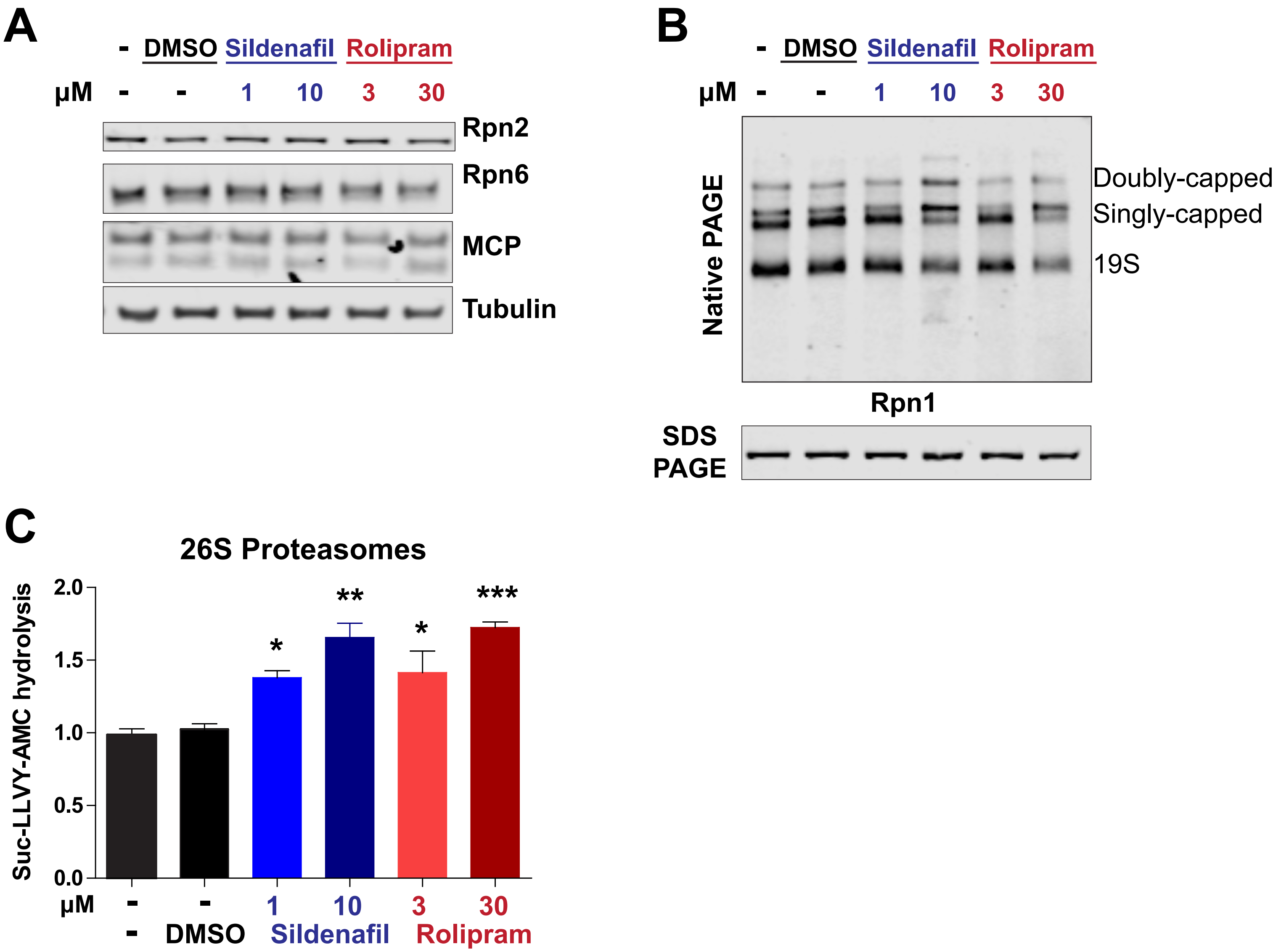

Supplemental Figure 9: Sildenafil and rolipram treatment of zebrafish larvae activates proteasomal activity without changing the levels of proteasome subunits

A.) Levels of proteasome subunits in zebrafish larvae were not changed by 5 day treatment with sildenafil or rolipram. The lysates were analyzed by western blot with antibodies that detect 19S subunits Rpn2 and Rpn6 and the 20S subunits α1-7 (MCP). Representative western blots are shown from one of three experiments.

B.) The amount of assembled 26S proteasomes was not changed by 5 day treatment with 1 μM sildenafil or 3 μM rolipram. Lysates of zebrafish larvae were analyzed by native-PAGE and western blot with an antibody against the 19S subunit Rpn1. The same samples were also analyzed by SDS-PAGE and western blot for Rpn1 to evaluate loading. Representative western blots are shown.

C.) Raising cGMP or cAMP stimulates the peptidase activity of 26S proteasomes. Zebrafish larvae were treated for 5 days with sildenafil or rolipram and then 26S proteasomes were purified via the Ubl method. 26S proteasomes were purified from 3 groups of larvae per condition (20-25 larvae/group). n=3 proteasome purifications. Averages +/- SEM. One-way AVOVA with Dunnett multiple comparison test. *$p \leq 0.05$. **$p \leq 0.01$. ***$p \leq 0.001$.

Supplemental Figure 10

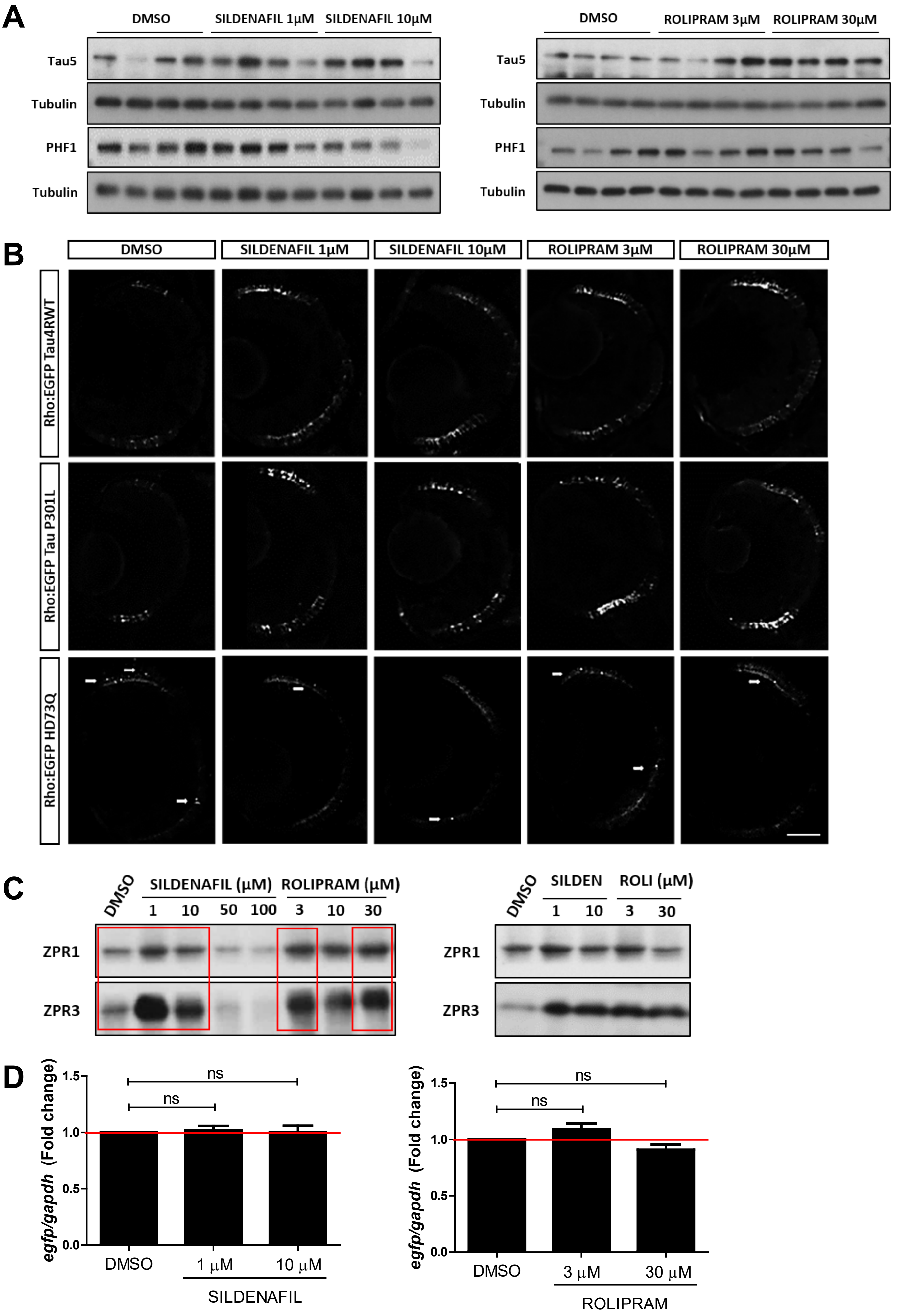

Supplemental Figure 10: Raising cGMP with sildenafil and raising cAMP with rolipram has beneficial effects in zebrafish models of neurodegenerative diseases.

A.) The levels of hyper-phosphorylated tau relative to total tau in Dendra-tau-A152T fish were decreased after treatment with sildenafil (1 and 10 μM) or rolipram (3 and 30 μM) (n=10/group). Representative blots of total tau (Tau5), phosphorylated tau (PHF1) and tubulin in Dendra-tau-A152T treated with DMSO, sildenafil (1 and 10 μM) and rolipram (3 and 30 μM). Quantification of these western blots is shown in Figure 5C.

B.) Sildenafil and rolipram rescued photoreceptor degeneration in Rho::EGFP-TauWT and Rho::EGFP-TauP301L zebrafish larvae. Representative images of sections through the central retina used for the quantification of photoreceptors (n=16/group) shown in Figure 5E.

C.) Sildenafil (1 and 10 μM) or rolipram (3 and 30 μM) reduce the degeneration of rod photoreceptors in Rho::EGFP-TauWT and Rho::EGFP-TauP301L zebrafish larvae. Representative western blots for the major rod photoreceptor protein, rhodopsin (ZPR3) and the loading control (arrestin, ZPR1). Quantification shown in Figure 5F.

D.) Sildenafil or rolipram did not change the expression of transgenes under the control of the rhodopsin promoter. Rho::EGFP fish were treated with sildenafil or rolipram from 4 days post fertilization (d.p.f) until 9 d.p.f. and the expression levels of egfp and gapdh were measured at 9 d.p.f. by quantitative PCR. No differences in egfp expression were found between treated and the control larvae (n= 12/group).

SI References

1. Guo X*, et al.* (2016) Site-specific proteasome phosphorylation controls cell proliferation and tumorigenesis. *Nature cell biology* 18(2):202-212.
2. Djakovic SN (2012) Phosphorylation of Rpt6 regulates synaptic strength in hippocampal neurons. 32(15):5126-5131.
3. VerPlank JJS, Lokireddy S, Zhao J, & Goldberg AL (2019) 26S Proteasomes are rapidly activated by diverse hormones and physiological states that raise cAMP and cause Rpn6 phosphorylation. *Proceedings of the National Academy of Sciences of the United States of America*.
4. Dimauro I, Pearson T, Caporossi D, & Jackson MJ (2012) A simple protocol for the subcellular fractionation of skeletal muscle cells and tissue. *BMC research notes* 5:513.
5. Kuo CL, Collins GA, & Goldberg AL (2018) Methods to Rapidly Prepare Mammalian 26S Proteasomes for Biochemical Analysis. *Methods in molecular biology (Clifton, N.J.)* 1844:277-288.
6. VerPlank JJS, Lokireddy S, Feltri ML, Goldberg AL, & Wrabetz L (2018) Impairment of protein degradation and proteasome function in hereditary neuropathies. *Glia* 66(2):379-395.
7. Zhao J, Zhai B, Gygi SP, & Goldberg AL (2015) mTOR inhibition activates overall protein degradation by the ubiquitin proteasome system as well as by autophagy. *Proceedings of the National Academy of Sciences of the United States of America* 112(52):15790-15797.
8. Kim HT, Collins GA, & Goldberg AL (2018) Measurement of the Multiple Activities of 26S Proteasomes. *Methods in molecular biology (Clifton, N.J.)* 1844:289-308.
9. Bodnar NO & Rapoport TA (2017) Molecular Mechanism of Substrate Processing by the Cdc48 ATPase Complex. *Cell* 169(4):722-735.e729.
10. VerPlank JJS & Goldberg AL (2018) Exploring the Regulation of Proteasome Function by Subunit Phosphorylation. *Methods in molecular biology (Clifton, N.J.)* 1844:309-319.
11. Kimmel CB, Ballard WW, Kimmel SR, Ullmann B, & Schilling TF (1995) Stages of embryonic development of the zebrafish. *Developmental dynamics : an official publication of the American Association of Anatomists* 203(3):253-310.
12. Moreau K*, et al.* (2014) PICALM modulates autophagy activity and tau accumulation. *Nature communications* 5:4998.
13. Williams A*, et al.* (2008) Novel targets for Huntington's disease in an mTOR-independent autophagy pathway. *Nature chemical biology* 4(5):295-305.
14. Lopez A*, et al.* (2017) A152T tau allele causes neurodegeneration that can be ameliorated in a zebrafish model by autophagy induction. *Brain : a journal of neurology* 140(4):1128-1146.
15. Scott EK*, et al.* (2007) Targeting neural circuitry in zebrafish using GAL4 enhancer trapping. *Nature methods* 4(4):323-326.
